# Functional Characterization and Conditional Regulation of the Type VI Secretion System in *Vibrio fluvialis*

**DOI:** 10.3389/fmicb.2017.00528

**Published:** 2017-03-30

**Authors:** Yuanming Huang, Pengcheng Du, Meng Zhao, Wei Liu, Yu Du, Baowei Diao, Jie Li, Biao Kan, Weili Liang

**Affiliations:** ^1^State Key Laboratory of Infectious Disease Prevention and Control, National Institute for Communicable Disease Control and Prevention, Chinese Center for Disease Control and PreventionBeijing, China; ^2^Collaborative Innovation Centre for Diagnosis and Treatment of Infectious DiseasesHangzhou, China; ^3^Beijing Key Laboratory of Emerging Infectious Diseases, Institute of Infectious Diseases, Beijing Ditan Hospital, Capital Medical UniversityBeijing, China

**Keywords:** Type VI secretion system, Hcp, VasH, T6SS, bacterial killing, *Vibrio fluvialis*

## Abstract

*Vibrio fluvialis* is an emerging foodborne pathogen of increasing public health concern. The mechanism(s) that contribute to the bacterial survival and disease are still poorly understood. In other bacterial species, type VI secretion systems (T6SSs) are known to contribute to bacterial pathogenicity by exerting toxic effects on host cells or competing bacterial species. In this study, we characterized the genetic organization and prevalence of two T6SS gene clusters (VflT6SS1 and VflT6SS2) in *V. fluvialis*. VflT6SS2 harbors three “orphan” *hcp-vgrG* modules and was more prevalent than VflT6SS1 in our isolates. We showed that VflT6SS2 is functionally active under low (25°C) and warm (30°C) temperatures by detecting the secretion of a T6SS substrate, Hcp. This finding suggests that VflT6SS2 may play an important role in the survival of the bacterium in the aquatic environment. The secretion of Hcp is growth phase-dependent and occurs in a narrow range of the growth phase (OD_600_ from 1.0 to 2.0). Osmolarity also regulates the function of VflT6SS2, as evidenced by our finding that increasing salinity (from 170 to 855 mM of NaCl) and exposure to high osmolarity KCl, sucrose, trehalose, or mannitol (equivalent to 340 mM of NaCl) induced significant secretion of Hcp under growth at 30°C. Furthermore, we found that although VflT6SS2 was inactive at a higher temperature (37°C), it became activated at this temperature if higher salinity conditions were present (from 513 to 855 mM of NaCl), indicating that it may be able to function under certain conditions in the infected host. Finally, we showed that the functional expression of VflT6SS2 is associated with anti-bacterial activity. This activity is Hcp-dependent and requires *vasH*, a transcriptional regulator of T6SS. In sum, our study demonstrates that VflT6SS2 provides *V. fluvialis* with an enhanced competitive fitness in the marine environment, and its activity is regulated by environmental signals, such as temperature and osmolarity.

## Introduction

*V. fluvialis*, a halophilic Gram-negative bacterium, is an emerging foodborne pathogen of increasing public health concern. It was originally isolated in 1975 from a patient suffering from severe diarrhea and was called “group F Vibrio” and “EF-6 Vibrio” by different research groups. The name *V. fluvialis* was proposed later by Lee et al. (1981). Since its discovery, the microbe has been implicated in both outbreaks and sporadic cases of diarrhea (Huq et al., 1980; Bellet et al., 1989; Klontz and Desenclos, 1990; Srinivasan et al., 2006; Bhattacharjee et al., 2010; Chowdhury et al., 2012), as well as various extra-intestinal infections (Huang and Hsu, 2005; Ratnaraja et al., 2005; Lai et al., 2006; Liu et al., 2011). Infection by *V. fluvialis* is most common in infants, children, and young adults. There has recently been an increase in the isolation rate from diarrheal patients, and multidrug-resistant clinical isolates of *V. fluvialis* have been reported (Ahmed et al., 2005; Srinivasan et al., 2006; Chowdhury et al., 2011, 2012). The clinical symptoms of *V. fluvialis* gastroenteritis are similar to those of *Vibrio cholerae*, except for the occurrence of bloody stool in *V. fluvialis* infection, which is a notable difference from cholera (Huq et al., 1980; Allton et al., 2006).

Despite increasing public health concern about *V. fluvialis*, knowledge about its microbiological characteristics, virulence factors, environmental fitness, and the epidemiology of infection is still limited (Igbinosa and Okoh, 2010; Liang et al., 2013). Many pathogenic factors, such as proteases, cytolysins, Chinese hamster_ovary (CHO) cell elongation factor, CHO cell-killing factor, enterotoxin-like substance, lipase, and hemolysin have been isolated and characterized, but their precise roles in the clinical manifestations and the pathogenicity of the bacterium remain to be explored (Lockwood et al., 1982; Chikahira and Hamada, 1988; Miyoshi et al., 2002; Kothary et al., 2003; Cabrera Rodríguez et al., 2005; Igbinosa and Okoh, 2010). Of the many virulence factors, hemolysin was thought to be of greatest importance. It was reported that hemolysin of *V. fluvialis* (VFH) formed larger pores in the erythrocyte membrane than other *Vibrio* hemolysins, including *V. cholerae* and *Vibrio vulnificus* (Han et al., 2002; Igbinosa and Okoh, 2010). Our previous study demonstrated that VFH contributes to the pathogenicity of *V. fluvialis* by inducing IL-1β secretion through the activation of the NLRP3 inflammasome (Song et al., 2015). We also showed that quorum sensing in *V. fluvialis* positively regulates the production of hemolysin and extracellular protease and affects the cytotoxic activity against epithelial tissue cultures (Wang et al., 2013).

The type VI secretion system (T6SS) is a newly discovered contact-dependent protein secretion system. Although the existence of T6SS had been postulated for more than a decade before, the T6SS was first functionally identified in O37 *V. cholerae* and *Pseudomonas aeruginosa* by the Mekalanos group in 2006 (Mougous et al., 2006; Pukatzki et al., 2006). In *V. cholerae*, it was demonstrated that the extracellular export of hemolysin-coregulated protein (Hcp) and three related valine-glycine repeat protein G (VgrG) proteins were required for the cytotoxicity of the organism in amoebae and macrophages (Pukatzki et al., 2006). In *P. aeruginosa*, HSI-I T6SS mediated the export of Hcp1, and evoked an immune response in cystic fibrosis (Mougous et al., 2006). Subsequent to these two landmark publications, numerous follow-up studies have demonstrated that T6SS is involved in the virulence and environmental competitive fitness of various bacterial species, such as *Burkholderia* species (Schell et al., 2007; Shalom et al., 2007), *Edwardsiella tarda* (Zheng and Leung, 2007), *Agrobacterium tumefaciens* (Wu et al., 2012), *Vibrio parahaemolyticus* (Yu et al., 2012), *Aeromonas hydrophila* (Suarez et al., 2008), *Citrobacter freundii* (Liu et al., 2015), *Vibrio alginolyticus* (Sheng et al., 2012; Salomon et al., 2015), and *Burkholderia cenocepacia* (Aubert et al., 2016). A genome-level survey revealed that more than 25% of genome-sequenced Gram-negative species contain T6SS gene clusters (Bingle et al., 2008). Although there are variations in their genetic contents and organization, 13 core T6SS genes have been recognized (Boyer et al., 2009). The T6SS secretion apparatus is structurally and functionally analogous to a contractile T4 bacteriophage tail, but has a reversed orientation (Leiman et al., 2009). A working model of the T6SS proposes that an intracellular Hcp nanotube, with a spike-like structure composed of a trimer of VgrG and PAAR (proline, alanine, alanine, arginine) motif-containing proteins at its top, is pushed through the envelope of the predator cell and into an adjacent target cell following the secretion of Hcp, VgrG, and other effectors (Shneider et al., 2013; Ho et al., 2014). This ejection process is powered by the contraction of the dynamic VipA/VipB sheath surrounding the Hcp nanotube (Basler et al., 2012). The T6SS is tightly regulated and has crosstalk with other regulatory systems, including the competence regulon, quorum sensing, and stress response (Ishikawa et al., 2009; Weber et al., 2009; Borgeaud et al., 2015).

We recently reported the draft genome of a clinically-isolated strain of *V. fluvialis*, 85003, and the results of a sequence analysis which revealed the presence of homologs of T6SS (Lu et al., 2014). However, questions about the genetic organization, functions, and regulation of these homologs remain unanswered. In this study, we aimed to further characterize the genetic contents and organization, functions, and the environmental conditions that control the expression of *V. fluvialis* T6SS. We found that there are two T6SSs in strain 85003, VflT6SS2 (*V. fluvialis* T6SS2), and VflT6SS1 (*V. fluvialis* T6SS1), with the former more prevalent than the latter in different *V. fluvialis* isolates. The genetic contents and organization of VflT6SS2 are highly homologous to the T6SS of *V. cholerae*, but contains three differently located “orphan” *hcp*-*vgrG* modules in distinct locations. Detection of Hcp expression and secretion revealed that VflT6SS2 is functionally activated under low (25°C) and warm (30°C) temperatures, but is inactive at the higher temperature (37°C). The secretion of Hcp is growth phase-dependent and only occurs at certain parts of the growth phase at 30°C. Osmolarity positively regulates the VflT6SS2 activity. Increasing the salinity (from 170 to 855 mM of NaCl) and exposure to high osmolarity KCl, sucrose, trehalose, or mannitol (equivalent to 340 mM NaCl) induced the secretion of Hcp. Furthermore, VflT6SS2 could also be activated at 37°C if higher salinity conditions were present (513 ~ 855 mM NaCl). In addition, a bacterial killing assay demonstrated that functional expression of VflT6SS2 has anti-bacterial activity which is dependent on Hcp and requires the T6SS transcriptional regulator, VasH.

## Materials and methods

### Bacterial strains, culture conditions, and plasmids

The bacterial strains and plasmids used in this study are listed in Table 1. The wild-type (WT) *V. fluvialis* 85003 and its derivative, as well as other *V. fluvialis* environmental and clinical isolates, were routinely grown in Luria-Bertani (LB) broth (pH 7.4) containing 170 mM NaCl at 30°C unless specifically indicated. *E. coli* SM10 λ*pir* and Rosetta (DE3) were routinely cultured at 37°C. Culture media were supplemented with ampicillin (Amp, 100 μg/ml), streptomycin (Sm, 100 μg/ml), rifampicin (Rfp, 50 μg/ml), kanamycin (Km, 50 μg/ml), tetracycline (Tc, 10 μg/ml for *E. coli*, 2.5 μg/ml for *V. fluvialis*), chloramphenicol (Cm, 10 μg/ml), or isopropyl-β-D-thiogalactopyranoside (IPTG), as required.

**Table 1:** **Strains and plasmids used in this study**.

**Strain/plasmid**	**Characteristics**	**References/sources**
***E. coli***		
SM10λ*pir*	*thi thr leu tonA lacY supE recA*::RP4-2Tc::Mu (λ*pir*R6K), Km^R^	Mekalanos Laboratory (Harvard Medical School)
Rosetta(DE3)	F^−^*ompT hsdS*_B_ (${\text{r}}_{\text{B}}^{-}$${\text{m}}_{\text{B}}^{-}$) *gal dcm*(DE3) pRARE^2^, Cm^R^	Laboratory stock
MG1655	K-12 F^−^ λ^−^*ilvG*^−^*rfb-50 rph-1*, Rfp^R^	Laboratory stock
***V. fluvialis***		
85003	*V. fluvialis*, wild type, Sm^R^	Lu et al., 2014
VF54	*V. fluvialis*, wild type, environmental isolate	This study
VF42	*V. fluvialis*, wild type, clinical isolate	This study
CICC21612	*V. fluvialis*, wild type, clinical isolate	Liang et al., 2013
Δ*vasH*	85003, Δ*vasH*	This study
Δ*tssD*2a	85003, Δ*tssD*2*_*a	This study
Δ*tssD*2b	85003, Δ*tssD*2*_*b	This study
Δ*tssD*2ab	85003, Δ*tssD*2*_*a, Δ*tssD*2*_*b	This study
Δ*tssDI*2c	85003, Δ*tssD*2*_*c-*tssI*2*_*c	This study
**PLASMID**		
pCVD442	Suicide vector containing R6K *ori, sacB*, Amp^R^	Laboratory stock
pWM91	Suicide vector containing R6K ori, *sacB, lacZα*; Amp^R^	Laboratory stock
pSRKTc	Broad-host-range vector containing *lac* promoter, *lacI*^q^, *lacZ*α, Tet^R^	Khan et al., 2008
pET30a(+)	expression vector containing pBR322 *ori*, f1 *ori, lacI*; Kan^R^	Novagen
pCVD-VFΔ*vasH*	1.79 kb *Sal*I-*Sac*I Δ*vasH* fragment of *V. fluvialis* in pCVD442	This study
pCVD-Δ*tssDI2_c*	1.69 kb *Sal*I-*Sac*I Δ*tssD_c*-*tssI2_c* fragment of *V. fluvialis* in pCVD442	This study
pWM-Δ*tssD*2*_a*	1.74 kb *BamH*I-*Sma*I Δ*tssD*2*_a* fragment of *V. fluvialis* in pWM91	This study
pWM-Δ*tssD*2*_*b	1.72 kb *Not*I-*Sma*I Δ*tssD*2*_b* fragment of *V. fluvialis* in pWM91	This study
pSR*vasH*	1.596 kb *vasH* ORF of *V. fluvialis* in pSRKTc	This study
pET30a-*hcp*	513 bp *Nco*I-*Xho*I fragment containing the coding sequence of *V. fluvialis hcp* in pET30a(+)	This study

### Determination of *hcp*-*vgrG* alleles and prevalence of T6SSs

To determine the sequence of the 3' end of VflT6SS2, primers vfu-vasL-up2153177 and vfu-rbsD-dn2158250 were designed according to the corresponding sequence of *Vibrio furnissii* NCTC11218. Similarly, primer pairs vfuA01956-up/vfuA01959-dn and vfuB01009-up/vfuB01011-dn were designed to amplify the *hcp*-*vgrG* alleles in 85003. The polymerase chain reaction (PCR) was performed with TranStart FastPfu Fly DNA polymerase (Transgen Biotech, China) with 85003 genomic DNA used as a template and the products were used for commercial sequencing.

To determine whether T6SS genetic loci are prevalent in isolates of *V. fluvialis*, PCR was performed with water-boiled templates of isolates to detect the presence of multiple chromosomal fragments specific for each T6SS gene cluster. For VflT6SS1, fragments covering *tssC*1 (*impC*)-*impD, impD*-*tssF*1 (*impG*), *tssF*1 (*impG*)-*tssG*1 (*impH*), *tssH*1, *tssH*1 (*clpB*)-*tagH*1 (*impI*), *tagH*1 (*impI*)-*tssK*1 (*impJ*), and *tssL*1 (*impK*)-*tssM*1 (*impL*) were respectively amplified by using primer pairs impC/impD-F and impC/impD-R, impD/impG-F, and impD/impG-R, impG/impH-F and impG/impH-R, clpB-F/clpB-R, clpB/impI-F and clpB/impI-R, lip/impJ-F, and lip/impJ-R, and impK/impL-F and impK/impL-R. For VflT6SS2, fragments covering *tssC*2 (*vipB*)-*tssF*2 (*vasA*), *tssG*2 (*vasB*)-*tagH2* (*vasC*), *tssJ*2 (*vasD*)-*tssK*2 (*vasE*), *tssL*2 (*vasF*)-*tssH*2 (*vasG*), *vasH-vasI, vasI*-*tssA*2 (*vasJ*), and *tssM*2 (*vasK*)-*vasL* were separately amplified with primer pairs vipB/vasA-F and vipB/vasA-R, vasB/vasC-F and vasB/vasC-R, vasD/vasE-F, and vasD/vasE-R, vasF/vasG-F and vasF/vasG-R, vasH/vasI-F and vasH/vasI -R, vasI/vasJ-F, and vasI/vasJ-R, and vasK/vasL-F and vasK/vasL-R. The oligonucleotide primers used are listed in Table 2.

**Table 2:** **Primers used in this study**.

**Primer**	**Oligonucleotide sequence (5′-3′)**
vfu-vasL-up2153177	CGTTGGAAGATTTTGCTGTG
vfu-rbsD-dn2158250	GTGAGGGCTAAATCAATACG
vfuA01956-up	CCGCCATTTTCGCTTACG
vfuA01959-dn	GCTTGATCCCAACCGCAG
vfu_B01009-up	CATTCAGGAAGATCCAGT
vfu_B01011-dn	GCTGCCAATCAGTTCCTA
impC/impD-F	AGCAATTTGAAGACGCACCT
impC/impD-R	GGCGAGAAAAATCAGGATGA
impD/impG-F	CAATCGCATCAACTGGTTTG
impD/impG-R	GCAACACCGCAATCACATAC
impG/impH-F	TGGACGAAGTCAACGAAGTG
impG/impH-R	ACACAGAAGGCGCTTGAGTT
clpB-F	TGTCCAAAGAAACGCATCTG
clpB-R	GAACACCATGGGCTCTTCAT
clpB/impI-F	ACGTCCGGAAAATGACGAT
clpB/impI-R	TAAGGTGTAGGCCCCAATCA
lip/impJ-F	TTCTGGCTGGGGTAACAAAG
lip/impJ-R	AGTTCAGCGGAAAAGCTCAC
impK/impL-F	AGCTCTACGCTCTGGGTTGA
impK/impL-R	ACTCGTCGTGCTGCTTGAG
vipB/vasA-F	CACCGATTTTGGCAGTGCTT
vipB/vasA-R	GCTGCAAAGCAAGATCCCAA
vasB/vasC-F	AAAACgCACCATCAATCgAgACA
vasB/vasC-R	CTggTgATgTACgTgCTTACAAC
vasD/vasE-F	AgATTgCgAATTAAgggACCAAA
vasD/vasE-R	TgTCCgCCgATTACgACCA
vasF/vasG-F	AAgCCTTCAATCATggCACTT
vasF/vasG-R	TTATCAggCgCTgTTggAAT
vasH/vasI-F	CCTgCgTCACTAACTCATTggA
vasH/vasI-R	TTCAACCgTTgCCgATgggA
vasI/vasJ-F	CTCTgCTCgACCTTgCgC
vasI/vasJ-R	CgTgTTgAACTggCgCTg
vasK/vasL-F	TTGGTGTGAAACCACGGGAT
vasK/vasL-R	CATCGGTGGAAGAGCTGAAG
vasH-F1-up-*Sal*I^*^	GCGTCGACGAATAAAACGGGAAGGCGAA
vasH-F1-dn	TGCAATGCTGTTATGACAAGATCGCTATAT
vasH-F2-up	CTTGTCATAACAGCATTGCATTTGAAAGCC
vasH-F2-up-*Sac*I^*^	CGAGCTCAGAAACGGTGGTGCAGCTTG
tssDI2c-F1-up-*Sal*I^*^	GCGTCGACGCAATTCAATGGAGCGCCAA
tssDI2c-F1-dn	ATTTTCATGA TGGCATCGTTTTTCCTTTAG
tssDI2c-F2-up	AACGATGCCA TCATGAAAATGACCCAGACT
tssDI2c-F2-dn-*Sac*I^*^	CGAGCTCTTACAATGACACGATTCGCC
tssD2a-F1-up-*BamH*I^*^	CGGGATCCGCCAGCTTTTTACATCGC
tssD2a-F1-dn	CGAGCGTAACTGCTCATTCCTTTCTAACTG
tssD2a-F2-up	GGAATGAGCAGTTACGCTCGAGCTGCGTTG
tssD2a-F2-dn-*Sma*I^*^	TCCCCCGGGAAACTGTAGTCTTGCAGC
tssD2b-F1-up-*Not*I^*^	AAATATGCGGCCGCGCAAGCGTTCTCTGATCT
tssD2b-F1-dn	CGAGCGTAACTGCTCATTCCTTTCTAACTG
tssD2b-F2-up	GGAATGAGCAGTTACGCTCGAGCTGCGTTG
tssD2b-F2-dn-*Sma*I^*^	TCCCCCGGGTCTGGCTCACCTCCGATT
VF-hcp-up-*Nco*I^*^	TGTCCATGGGCCCAACTCCATGTTATATCTCTATCG
VF-hcp-dn-*Xho*I^*^	AGACTCGAGCGCTTCGATTGGTTTACGCCA
vasH-For-*Hind*III^*^	CCCAAGCTTTCATAACGCCTTGATCTC
vasH-Rev-*Nde*I^*^	GGAATTCCATATGAGTAACTGGCTCGCT
VF-recA-qPCR-up	ACCGAGTCAACGACGATAAC
VF-recA-qPCR-dn	TGATGAACTGCTGGTGTCTC
hcp-qPCR-F-com	TCGGCGATTCATTCGTT
hcp-qPCR-R-com	CAGTTCAACCGTCGTCATCT
VF-vasF-qPCR-F	CTGTGGCTCTTCCTCTTC
VF-vasF-qPCR-R	TTATCAGTGCTTGGTGTTG
VF-vasK-qPCR-F	ACATCCAACGCCAATACG
VF-vasK-qPCR-R	CAATCGCAGTGAAGACAAC
VF-vasH-qPCR-F	GGTAATCGGATACTGGAAC
VF-vasH-qPCR-R	CATGTCAACTTGCTGGAT
VF-tssD1-qPCR-F	TGTCGGTCACTCGTAACTC
VF-tssD1-qPCR-R	TCAGAACCAGCACCATCAC
vflB629-qPCR-F	GGTGGAAGTGTCTGGATGG
vflB629-qPCR-R	TGGCTCAGGTTGGTATGC
vflB643-qPCR-F	TGAACAACCGAGTGGCGAATATC
vflB643-qPCR-R	CAGTTGGCGGAACGAGGATTG
vflB631-qPCR-F	CGAGCCGAATATCCGTCTG
vflB631-qPCR-R	TTCCTGCCGTTCCAACAC
vflB647-qPCR-F	TTGCGGAAGTGATCTCTG
vflB647-qPCR-R	TTACGGCTGTCGGTTAAG

* *The underlined text indicates the restriction enzyme sites*.

### Construction of mutants and complementation plasmids

The in-frame deletion mutants Δ*vasH*, Δ*tssD*2a, Δ*tssD*2b, and Δ*tssDI*2c were constructed by allelic exchange using the clinical strain 85003 as a WT precursor as described previously (Wu et al., 2015). We used Δ*tssD*2a as a precursor to construct the double mutant, Δ*tssD*2ab. Briefly, the chromosomal fragments containing upstream and downstream nucleotide sequences of target genes were respectively amplified using the corresponding primers listed in Table 2 and then were stitched together by overlapping PCR. The 1.79 kb Δ*vasH* and 1.69 kb Δ*tssD_c*-*tssI2_c* fragments were individually cloned at *Sal*I-*Sac*I sites in a pCVD442 suicide plasmid to yield pCVD-VFΔ*vasH* and pCVD-Δ*tssDI2_c*. The 1.74 kb Δ*tssD*2*_a* and 1.72 kb Δ*tssD*2*_b* fragments were cloned at *BamH*I-*Sma*I sites and *Not*I-*Sma*I sites in the suicide plasmid pWM91 to yield pWM-Δ*tssD*2*_a* and pWM-Δ*tssD*2*_*b, respectively. The resulting recombinant suicide plasmids were conjugated into *V. fluvialis* from *E. coli* SM10λ*pir* and transconjugants were selected on LB media containing Amp and Sm. The transconjugants were counter-selected by growing them on media containing 15% sucrose. Sucrose-resistant colonies were tested for Amp sensitivity, and target deletion was identified by PCR and confirmed by DNA sequencing.

The *vasH* expression plasmid was constructed for complementation test. Primers vasH-For-*Hind*III and vasH-Rev-*Nde*I were used to amplify the ORF of *vasH* with PrimeSTAR® HS DNA Polymerase (TaKaRa, Dalian, China) using WT 85003 genomic DNA as a template. The 1,596 bp PCR product with blunt ends was digested with *Nde*I and cloned at *Nde*I and *Sma*I sites in pSRKTc to generate pSR*vasH*, which expresses *vasH* from the *lac* promoter with the induction of IPTG. The pSR*vasH* was mobilized into Δ*vasH* mutant from *E. coli* SM10λ*pir* by conjugation, and the expression vector pSRKTc without an insert was used as a negative control.

### Anti-Hcp polyclonal antiserum preparation

An anti-Hcp polyclonal antibody was raised in rabbits using the following method: First, the *hcp* gene was amplified by PCR using primer pair VF-hcp-up-*Nco*I/VF-hcp-dn-*Xho*I and was cloned into pET30a (+) using *Nco*I and *Xho*I restriction enzyme sites. His-tagged Hcp expression was induced by IPTG in *E. coli* Rosetta (DE3) host cells and was purified by Ni-IDA affinity chromatography (Novagen) under native conditions according to the manufacturer's instructions. The purified Hcp protein was used for immunization of rabbits by Wuhan Abzome Biotechnology, China. The polyclonal antiserum was purified with an antigen affinity purification procedure before use.

### Analysis of T6SS protein production and secretion

Overnight cultures of bacterial strains were diluted to 1:100 in 20 mL of fresh LB containing 170 mM NaCl with shaking, and were incubated to an optical density at 600 nm (OD_600_) of 1.5. In the *vasH* complementation test, overnight cultures of Δ*vasH* mutants containing plasmid pSR*vasH* (Δ*vasH/*pSR*vasH*) or pSRKTc (Δ*vasH/*pSRKTc) were grown in the above LB with tetracycline to an OD_600_ of 0.5. Each culture was then divided in half. One half was induced by the addition of IPTG (final concentration of 0.5 mM), and the other half (uninduced) was used as a control. The cultures were continually incubated until they reached an OD_600_ of 1.5 at 30°C with shaking.

Cells were pelleted at high speed in a tabletop microcentrifuge for 5 min. Equal volumes of supernatants were filtered through 0.22 μm low protein-binding polyvinylidine fluoride syringe filters (Millipore), and then a 1/100 volume of 2% sodium deoxycholate was added, and the samples were incubated for 30 min on ice. Proteins were precipitated with 10% trichloroacetic acid (TCA) for 30 min on ice, pelleted by centrifugation at 12,000 rpm for 20 min at 4°C, and washed twice with ice-cold acetone to remove residual TCA. Protein precipitates were resuspended in 40 μl SDS-PAGE loading buffer (10 mM Tris-HCl, pH 8.0; 50 mM DTT; 1% SDS; 10% glycerol; 0.08% bromophenol blue) and boiled for 10 min. Cell pellets were resuspended in PBS and the cell density was adjusted to 3.5 McFarland standards. Then pellets from 1 ml aliquots of above samples were suspended with 150 μl of SDS-PAGE lysis buffer and boiled for 10 min. Protein samples were separated by SDS-PAGE (12%), transferred onto PVDF membranes, and analyzed by Western blotting using a rabbit anti-Hcp polyclonal antibody and an anti-*E. coli* CRP antibody (BioLegend, USA). The secondary antibodies used were horseradish peroxidase (HRP)-conjugated goat anti-rabbit and goat anti-mouse antibodies. Proteins were visualized using an Enhanced Chemiluminescence system (TaKaRa, Dalian, China). Each immunoblot experiment was repeated at least two times.

### RNA extraction and qRT-PCR

Total RNA extraction and quantitative reverse transcription (qRT)-PCR were performed as described previously (Wu et al., 2015). In brief, strain 85003 was grown to OD600 1.5 under the indicated conditions with agitation. Three independent samples were tested in triplicate. A control mixture using total RNA as a template was added in each reaction to exclude the effects of chromosomal DNA contamination. Relative expression values (R) were calculated using the equation *R* = 2^−(Δ*Cq target* − Δ*Cq reference*)^, where Cq is the fractional threshold cycle. We used *recA* mRNA as a reference. The following primer combinations were used: VF-recA-qPCR-up and VF-recA-qPCR-dn for *recA*; hcp-qPCR-F-com and hcp-qPCR-R-com for *tssD*2 (*hcp*); VF-vasF-qPCR-F and VF-vasF-qPCR-R for *tssL*2 (*vasF*); VF-vasH-qPCR-F and VF-vasH-qPCR-R for *vasH*; VF-vasK-qPCR-F and VF-vasK-qPCR-F for *tssM*2 (*vasK*); VF-tssD1-qPCR-F and VF-tssD1-qPCR-R for *tssD*1; vflB629-qPCR-F and vflB629-qPCR-R for *tssM*1; vflB643-qPCR-F and vflB643-qPCR-R for *tssA*1; vflB631-qPCR-F and vflB631-qPCR-R for *tssK*1 and vflB647-qPCR-F and vflB647-qPCR-R for *rhs*. Detailed primer sequence information is listed in Table 2.

### Bacterial killing assay

*V. fluvialis* predator strains 85003 and Δ*vasH*, and *E. coli* prey MG1655, were grown overnight on LB agar containing 170 mM NaCl at 30°C. Bacterial lawns were resuspended and normalized to 1.5 McFarland standards in PBS and mixed at a 9:1 ratio (predator: prey) in triplicates. A total of 10 μl of the mixtures were spotted on LB plates with 340 mM NaCl and incubated at 30°C for 5 h. The colony forming units (CFU) per milliliter of the attacker and prey in the mixtures (0 h) were determined by plating 10-fold serial dilutions on streptomycin- and rifampicin-containing agar plates, respectively. Bacterial spots were harvested from LB plates after 5 h, and the CFU per milliliter of the surviving attacker and prey (5 h) were determined on selective media plates as described above.

In the *vasH* complementation assay, IPTG was added to LB plates at a final concentration of 1 mM to induce *vasH* expression. *V. fluvialis* strain Δ*vasH/*pSR*vasH* was grown overnight at 30°C on tetracycline-containing LB agar with 170 mM NaCl in the presence or absence of IPTG. The mixtures with prey were prepared as described above and spotted on LB plates containing 340 mM NaCl with and without IPTG. Strain Δ*vasH/*pSRKTc was used as a control.

### Nucleotide sequences and accession numbers

The sequences of VflT6SS1, VflT6SS2, and the three *hcp-vgrG* homologs from *V. fluvialis* 85003 were deposited in the NCBI database under accession numbers KY319183, KY319184, KY319185, KY319186, and KY319187, respectively. The genomic sequences of *V. furnissii* NCTC11218 (accession numbers NC_016602.1 and NC_016628.1), *V. fluvialis* 33809 (accession numbers CP014034 and CP014035), *V. cholerae* N16961 (accession numbers NC_002505.1 and NC_002506.1) and *V. splendidus* LGP32 (accession numbers NC_011753.2 and NC_011744.2) were downloaded from the NCBI database.

### DNA and protein sequence analysis

The comparative analysis of T6SS sequences from *V. fluvialis* strains 85003 and 33809, *V. cholerae* N16961, *V. furnissii* NCTC11218 and *V. splendidus* LGP32 were performed using the BLAST software with an e-value of 1e-2, and the alignments of >1 kilobase (kb) were kept. VasH protein sequence alignments were performed with the GENEDOC program.

## Results

### Genetic contents and organization of T6SS in *V. fluvialis*

Our previous draft genome analysis of *V. fluvialis* 85003 revealed the presence of putative homologs of T6SSs, which clustered and grouped into two genetic modules which are highly homologous to two separate regions on chromosome 2 of *V. furnissii* (Lu et al., 2014). In this study, we defined the detailed gene contents and organization of the two T6SSs, the flanking regions of the two T6SS clusters, and further identified two other “orphan” *hcp*-*vgrG* homologs by PCR with primers designed based on the sequence of *V. furnissii* NCTC11218.

**Figure 1:**
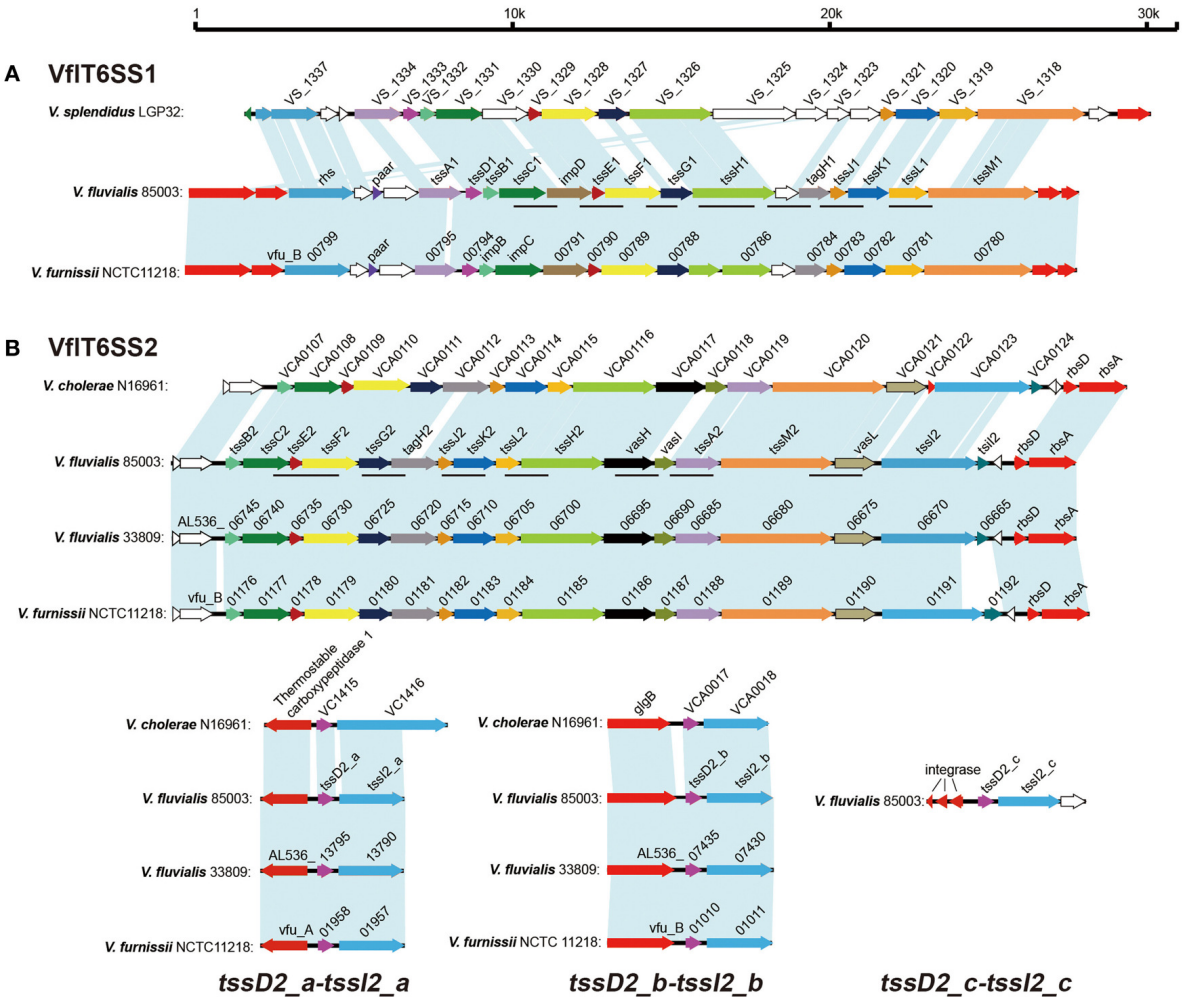
**Gene organization of the T6SS clusters in ***V. fluvialis*** in comparison with ***V. furnissii***, ***V. cholerae***, and ***V. splendidus*****. **(A)** Genes encoding VflT6SS1 in *V. fluvialis* 85003 were named according to the Tss nomenclature (*tssA*1, *tssB*1, *tssC*1, *tssD*1, *tssE*1, *tssF*1, *tssG*1, *tssH*1, *tssJ*1, *tssK*1, *tssL*1, *tssM*1, *tagH*1) or by their usual vernacular names (*paar, impD*, and *rhs*). Homologous genes are colored similarly. Open reading frames with unknown functions are shown in white. The lines below the colored genes indicate the loci amplified by PCR in the different isolates. **(B)** Genes encoding VflT6SS2 in *V. fluvialis* 85003 were names according to the Tss nomenclature (*tssA*2, *tssB*2, *tssC*2, *tssE*2, *tssF*2, *tssG*2, *tssH*2, *tssI*2, *tssJ*2, *tssK*2, *tssL*2, *tssM*2, *tagH*2) or vernacular names (*vasH, vasI, tsiI2*, and *vasL*). The 17 genes show high conservation with the well-characterized T6SS of *V. cholerae* N16961. The counterparts of *V. fluvialis* 33809 and *V. furnissii* NCTC11218 exhibit the identical gene organization in their respective chromosomes. The lines below the colored genes indicate the loci amplified by PCR in the different isolates. Three “orphan” *hcp-vgrG* alleles were designated *tssD*2_a-*tssI*2_a, *tssD*2_b-*tssI*2_b, and *tssD*2_c-*tssI*2_c, respectively. The homolog of *tssD*2_c-*tssI*2_c is absent in *V. fluvialis* 33809, *V. furnissii* NCTC11218, and *V. cholerae* N16961.

The cluster homologous to the *V. furnissii* vfu_B00799~vfu_B00780 was named VflT6SS1, and also shared partial synteny with the genetic region of VS_1337~VS_1318 of *V. splendidus*. In *V. furnissii*, three genes, vfu_B01185, vfu_B01189, and vfu_B01191, were predicted to encode T6SS-related subunits ClpB, IcmF-like protein and VgrG, respectively. In *V. splendidus*, only VS_1326 was annotated to encode ClpB. The VflT6SS1 is a locus of around 24 kb consisting of 19 open reading frames (ORFs) with tight intergenic spaces and the same gene orientation. Homologs of *V. splendidus* VS_1324 and VS_1325 were not found in either *V. furnissii* or *V. fluvialis*. We designated 13 out of the 19 ORFs as VflT6SS1 *tss*A1-M1 (Type Six Secretion A1-M1) following the proposed nomenclature for T6SS components (Shalom et al., 2007; Figure 1). These showed 82–100% and 27–79% amino acid identity with the corresponding homologs of *V. furnissii* and *V. splendidus*, respectively. The VflT6SS1 cluster contains the T6SS hallmark gene, *tssD* (*hcp*), *tssH* (*clpV/vasG*) encoding an ortholog of the ClpB ATPase, which is considered important for T6SS function, and two additional genes, *tssL* (*icmH*/*dotU*/*impK*) and *tssM* (*icmF*/*impL*), encoding homologs of T4SS stabilizing proteins (Boyer et al., 2009). Of note, the *hcp* of VflT6SS1 had only 40% nucleotide identity to the homologs in *V. cholerae* (VC1415 and VCA0017). To gain insight into the genetic background of VflT6SS1, its flanking regions were examined. The flanking sequence of 85003 VflT6SS1 was identical to that of *V. furnissii* NCTC 11218. The left junction of the sequences encodes a putative ABC-type transport periplasmic component and a MFS transporter, and the right junction possesses the sensor histidine kinase, BaeS. However, no homologous gene cluster of VflT6SS1 was found in *V. fluvialis* strain ATCC 33809, a newly whole-genome sequenced *V. fluvialis* isolate from Bangladesh.

The cluster homologous to the *V. furnissii* vfu_B01176~vfu_B01191 genomic region was named VflT6SS2. It spans 21.22 kb and contains 16 ORFs which display the same organization and gene orientation as the “core” gene cluster of *V. cholerae* T6SS (VCA0107~VCA0124). The major characteristics of the VflT6SS2 gene cluster of *V. fluvialis* strain 85003 and its most closely related orthologs in other species are summarized in Table 3.

**Table 3:** **Characteristics of VflT6SS2 components and the most closely related orthologs in other species**.

***V. fluvialis*** **85003**			**Homologs**	***V. cholerae* N16961**	***V. furnissii* NCTC11218**	***V. fluvialis* 33809**
**Gene**	**Size (bp)**	**COG**		**Gene ID (identity%)**	**Gene ID (identity%)**	**Gene ID (identity%)**
*tssB*2	510	3,516	*impB, vipA*	VCA0107 (87.88)	vfu_B01176 (96.47)	AL536_06745 (98.82)
*tssC*2	1,476	3,517	*impC, vipB*	VCA0108 (92.68)	vfu_B01177 (98.58)	AL536_06740 (98.37)
*tssE*2	438	3,518	*impF, vasS, hsiF*	VCA0109 (89.73)	vfu_B01178 (100.00)	AL536_06735 (99.31)
*tssF*2	1,770	3,519	*impG, vasA*	VCA0110 (84.92)	vfu_B01179 (98.47)	AL536_06730 (99.83)
*tssG*2	1,017	3,520	*impH, vasB*	VCA0111 (79.35)	vfu_B01180 (96.95)	AL536_06725 (99.70)
*tagH2*	1,479	3,456	*impI, vasC, fha*	VCA0112 (64.86)	vfu_B01181 (91.73)	AL536_06720 (97.76)
*tssJ*2	477	3,521	*lip, vasD*	VCA0113 (84.91)	vfu_B01182 (98.74)	AL536_06715 (98.11)
*tssK*2	1,335	3,522	*impJ, vasE*	VCA0114 (90.34)	vfu_B01183 (98.88)	AL536_06710 (97.30)
*tssL*2	774	3,455	*impK, vasF, icmH/dotU*,	VCA0115 (86.05)	vfu_B01184 (99.61)	AL536_06705 (100.00)
*tssH*2	2,613	0,542	*clpV, vasG*	VCA0116 (86.09)	vfu_B01185 (97.47)	AL536_06700 (96.79)
*vasH*	1,596	1,221	*sfa*	VCA0117 (73.82)	vfu_B01186 (96.11)	AL536_06695 (98.5)
*vasI*	660	–	*vasI*	VCA0118 (47.81)	vfu_B01187 (86.76)	AL536_06690 (95.91)
*tssA*2	1,401	3,515	*impA, vasJ*	VCA0119 (70.42)	vfu_B01188 (95.29)	AL536_06685 (98.72)
*tssM*2	3,546	3,523	*impL, icmF, vasK*	VCA0120 (85.19)	vfu_B01189 (98.48)	AL536_06680 (100)
*vasL*	1,278	3,515	*vasL*	VCA0121 (55.45)	vfu_B01190 (93.43)	AL536_06675 (98.35)
*tssI*2	3,039	3,501	*vgrG*	VCA0123 (54.03)	vfu_B01191 (73.74)	AL536_06670 (97.00)
*tsiI2*	384	–	*tsiV3*	VCA0124	vfu_B01192	AL536_06665 (94.51)
*tssD2_a*	519	3,157	*hcp*	VC1415 (95.38)	vfu_A01958 (99.42)	AL536_13795 (100.00)
*tssI2_a*	2,067	3,501	*vgrG*	VC1416 (44.58)	vfu_A01957 (96.37)	AL536_13790 (96.22)
*tssD2_b*	519	3,157	*hcp*	VCA0017 (95.38)	vfu_B01010 (99.42)	AL536_07435 (100.00)
*tssI2_b*	2,097	3,501	*vgrG*	VCA0018 (74.24)	vfu_B01011 (97.28)	AL536_07430 (97.14)
*tssD2_c*	519	3,157	*hcp*	–	–	–
*tssI2_c*	1,980	3,501	*vgrG*	–	–	–

–*, Unknown or does not exist*.

VflT6SS2 is located between the *usp* and *rsbD* genes in 85003, the same as in *V. cholerae* and *V. furnissii*, indicating its conserved location. Apart from the core gene cluster, *V. cholerae* T6SS has two copies of *hcp*-*vgrG* alleles encoded in two small auxiliary clusters (VCA0017~VCA0021 and VC1415~VC1421). In the 85003 draft genome, we only identified one copy of the *hcp*-*vgrG* homolog and the counterparts of vfu_B001191 and VCA0124 (*vgrG3*) from *V. furnissii* and *V. cholerae*, respectively, were missing in the originally aligned VflT6SS2 genetic locus. Sequence alignments showed that the two copies of *hcp*-*vgrG* modules exist in similar genetic locations in both *V. furnissii* and *V. cholerae*. Therefore, we reasoned that undiscovered *hcp*-*vgrG* copies likely existed in *V. fluvialis* 85003. *V. fluvialis* and *V. furnissii* are the genetically closest species among *Vibrionaceae*, with a mean BLASTp identity of 86.8% for the homologous genes (Lu et al., 2014). *V. furnissii* was originally regarded as an aerogenic biogroup of *V. fluvialis* and was later confirmed to be a separate species from *V. fluvialis* (Brenner et al., 1983). Using primer pairs vfu-vasL-up2153177/vfu-rbsD-dn2158250, vfuA01956-up/vfuA01959-dn, and vfuB01009-up/vfuB01011-dn, which were designed on the basis of the flanking sequences of *V. furnissii* vfu_B01191~vfu_B01192 and the two *hcp*-*vgrG* modules (vfu_A01957~vfu_A01958 and vfu_B01010~vfu_B01011, respectively), we amplified the corresponding regions of 85003 with long fragment PCR.

Primer pair vfu-vasL-up2153177/vfu-rbsD-dn2158250 yielded a 5093 bp fragment for *V. fluvialis* 85003. A sequence analysis of the fragment revealed a 3039 bp ORF which had 82 and 60% nucleotide identity to vfu_B001191 and VCA0123 (*vgrG*3), respectively. Additionally, a 384 bp ORF was predicted immediately downstream and has 4 bp overlap with the 3,039 bp ORF. This organizational feature was similar to VCA0123 and VCA0124, which were experimentally demonstrated to encode a *vgrG3* effector and its immunity effector, TsiV3, in *V. cholerae* (Dong et al., 2013). Thus, we named the two ORF *tssI*2 and *tsiI*2, respectively. A sequence analysis and alignment of the 3,145 and 3,878 bp PCR products of primer pair vfuA01956-up/vfuA01959-up and vfuB01009-up/vfuB01011-dn indicated that each encodes a copy of the *hcp*-*vgrG* homolog, which was different from the originally recognized *hcp*-*vgrG* allele. In order to distinguish them, we designated the newly- identified alleles as *tssD*2*_a*-*tssI*2*_a* and *tssD*2*_b*-*tssI*2*_b* (corresponding to *V. cholerae hcp*1-*vgrG*1 and *hcp*2-*vgrG*2, respectively), and the original one was named *tssD*2*_c*-*tssI*2*_c*. The three copies of TssD (Hcp) show high identity (99.42%) in their nucleotide sequences and have 95.35% amino acid identity to the well-characterized Hcp protein of *V. cholerae*. TssI2_a and TssI2_b display higher homology with up to 88% amino acid identity. TssI2_c shows 60 and 61% identity to TssI2_a and TssI2_b respectively. The corresponding sequences of these three *hcp*-*vgrG* alleles were deposited in GenBank under accession numbers KY319185, KY319186, and KY319187, respectively.

### Prevalence of *V. fluvialis* T6SS

In many species, T6SS gene clusters are located on pathogenicity islands or compositionally distinct regions of the genome indicating its horizontal acquisition and transfer. To better understand the gene content, sequence similarity, synteny, and the distribution of T6SS clusters in *V. fluvialis*, we carried out PCR screening of each T6SS locus in 34 different *V. fluvialis* isolates from both human and aquatic products from different provinces in China. The genetic loci of *tssC*1 (*impC*)-*impD, tssE*1 (*impF*)-*tssF*1 (*impG*), *tssF*1 (*impG*)-*tssG*1 (*impH*), *tssH*1, *tssH*1 (*clpB*)-*tagH*1 (*impI*), *tagH*1-*tssK*1 (*impJ*), and *tssL*1 (*impK*)-*tssM*1 (*impL*) within VflT6SS1 were all detected in seven out of 34 tested isolates (21% detection rate). The genetic loci of *tssC*2 (*vipB*)-*tssF*2 (*vasA*), *tssG*2 (*vasB*)-*tagH2* (*vasC*), *tssJ*2 (*vasD*)-*tssK*2 (*vasE*), *tssL*2 (*vasF*)-*tssH*2 (*vasG*), *vasH*-*vasI, vasI*-*tssA*2 (*vasJ*), and *tssM*2 (*vasK*)-*vasL* for VflT6SS2 were detected in 100, 97, 97, 100, 91, 94, and 94% of the isolates, respectively. These results revealed that in general, VflT6SS2 is more prevalent than VflT6SS1 in *V. fluvialis*. The different detection rates of VflT6SS2 component gene loci may indicate that there is variation in the gene composition of in the T6SS2 cluster or sequence variation from the corresponding primer annealing regions in different isolates. These variations need to be investigated in greater detail in future studies. Since we found that VflT6SS2 is highly prevalent in *V. fluvialis* strains, we first focused on the functional characterization of VflT6SS2 in *V. fluvialis* 85003.

### Determination of the functional expression of *V. fluvialis* VflT6SS2

The hallmark of a functional T6SS is the presence of Hcp and VgrG in the culture supernatant. Therefore, we examined the Hcp secretion in *V. fluvialis* cultures. To this end, we first cloned and expressed *hcp* in pET30a with induction by IPTG. The purified Hcp protein was used to prepare anti-Hcp polyclonal antiserum in rabbits, because there is no commercially-available antibody against this protein.

**Figure 2:**
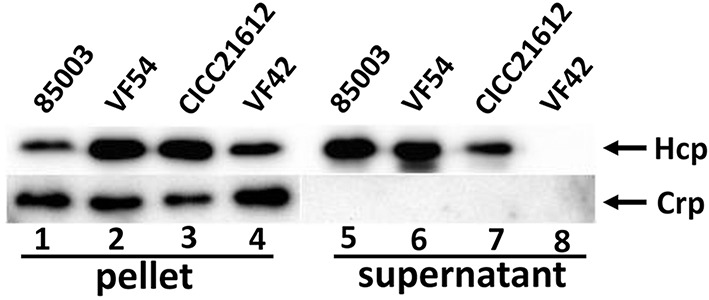
**Immunoblot analyses of VflT6SS2 Hcp in cell pellets and supernatants from ***V. fluvialis*** isolates**. The bacterial strains were grown in LB with 170 mM NaCl at 30°C to an OD_600_ of 1.5. SDS-PAGE and immunoblot analyses were performed using anti-Hcp and anti-Crp antibodies. Lanes 1–4, cell pellets; lanes 5–8, culture supernatants of *V. fluvialis* strains 85003, VF54, VF42, and CICC21612, respectively. The arrows show the immunoblot band to Hcp and Crp. The Crp protein is absent in the culture supernatants, indicating that the detection of Hcp in the supernatants was not a consequence of cell lysis.

To determine whether VflT6SS2 was functionally expressed, clinical isolates 85003, VF42, and CICC21612, and environmental isolate VF54, all of which contain the complete VflT6SS2 based on a PCR screen, were routinely cultured in LB with 170 mM NaCl at 30°C. The growth curves of VF42, VF54, and CICC21612 displayed consistent growth trends before entering a stationary growth phase, while 85003 grew slowly relative to these other strains (Figure S1). However, all four strains were in the logarithmic growth phase at an OD600 of 1.5. The cell pellets and culture supernatants of each strain at an OD_600_ of 1.5 were examined by immunoblot analyses using polyclonal anti-Hcp antiserum. As shown in Figure 2, a large amount of Hcp was detected in both the cell pellets and culture supernatants of 85003, CICC21612, and VF54, indicating the functional expression of T6SS2 in these *V. fluvialis* strains. Of note, the absence of the cytosolic Crp in the supernatants demonstrated that the bacteria were intact, and thus, Hcp was being actively secreted and not released by cell lysis. This control was routinely used in the subsequent Hcp secretion analysis. For strain VF42, Hcp was only detected in the cell pellets, indicating that, though Hcp was synthesized in these bacteria, it was not efficiently secreted into the supernatant. Taken together, our results demonstrated that functional T6SS exists in at least some *V. fluvialis* isolates.

It's should be noted that the apparent molecular weight of Hcp in the Western blot analysis (around 25 kDa) is quite different from the predicted size according to its ORF sequence (19 kDa). This inconsistency has also been observed in *V. cholerae* (Ishikawa et al., 2009) and *V. alginolyticus* (Sheng et al., 2012), and possible modification of two cysteine residues of Hcp at positions 5 and 22 was suggested to be responsible for the difference (Williams et al., 1996).

### Growth phase-dependent secretion of Hcp from *V. fluvialis* VflT6SS2

**Figure 3:**
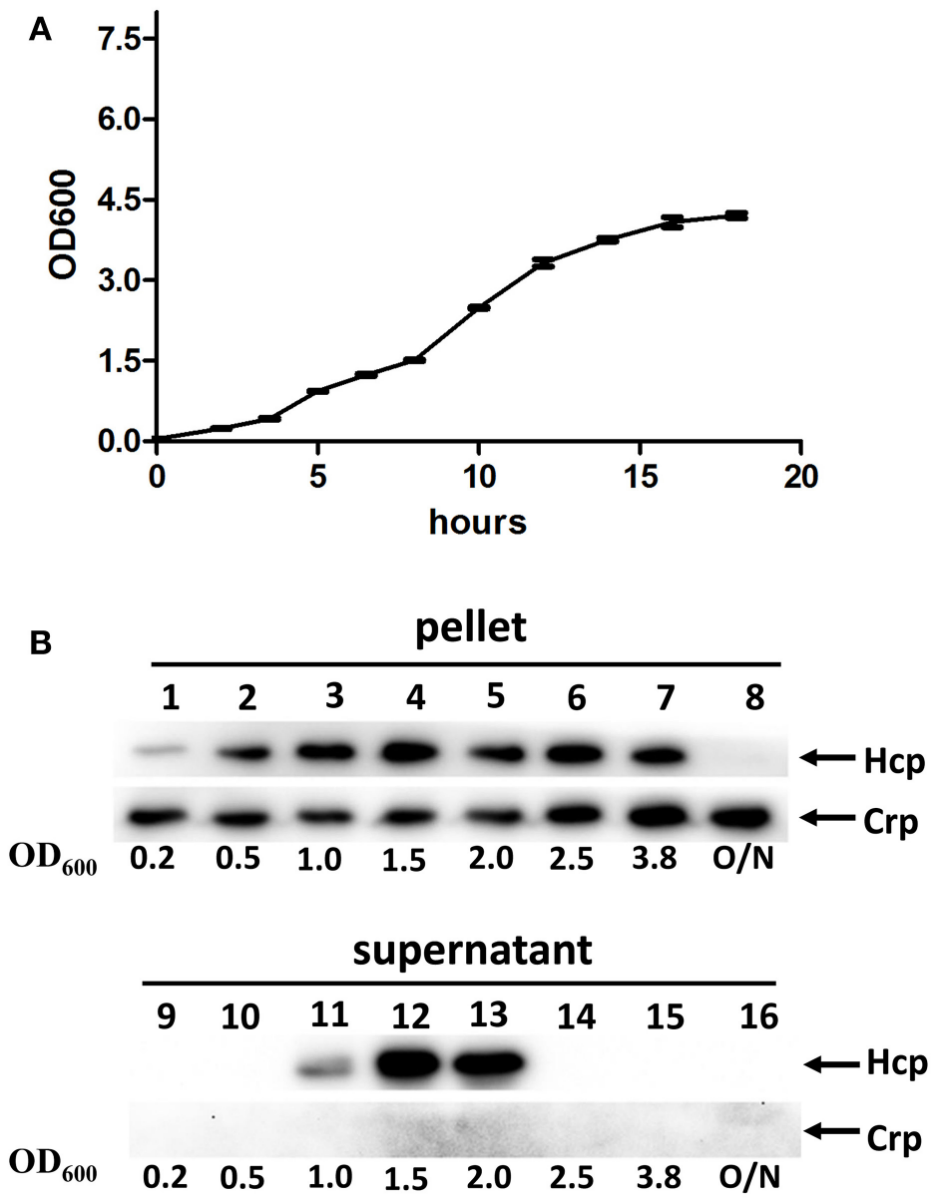
**VflT6SS2 Hcp levels in ***V. fluvialis*** 85003 at different parts of the growth phase. (A)** Growth curve of *V. fluvialis* strain 85003 incubated in LB media containing 170 mM NaCl at 30°C. Error bars indicate the standard deviation of three independent cultures. **(B)** Immunoblot analysis of Hcp expression. The *V. fluvialis* strain 85003 was grown in LB containing 170 mM NaCl at 30°C and the cell pellet and culture supernatant samples were taken at different parts of the growth phase (OD_600nm_ 0.2, 0.5, 1.0, 1.5, 2.0, 2.5, 3.8, and overnight). SDS-PAGE and immunoblot analyses were performed using anti-Hcp and anti-Crp antibodies. Lanes 1–8, cell pellets; lanes 9–16, culture supernatants. The arrows indicate the reaction bands of the Hcp and Crp proteins.

Following the confirmation of the functional expression of VflT6SS2 in *V. fluvialis*, we tested the effects of the bacterial growth phase on the expression and secretion of Hcp. Strain 85003 was grown in LB medium containing 170 mM NaCl at 30°C, and the protein levels of Hcp in the cell pellets and culture supernatants were measured at different growth points (i.e., OD_600_ 0.2, 0.5, 1.0, 1.5, 2.0, 2.5, 3.8, and overnight; Figures 3A,B). Hcp expression was detected in the cell pellets at all growth points except overnight, and the expression level at an OD_600_ of 0.2 was obviously lower than that of the other growth points (Figure 3B, lane 1). However, the secretion of Hcp was only detected at OD_600_ values of 1.0, 1.5, and 2.0, and the highest secretion level was observed at an OD_600_ of 1.5 (Figure 3B, lanes 11–13). Our results indicated that, although the Hcp was continuously synthesized at various OD_600_ values (except overnight culture), the active T6SS was only detected during a narrow window of the growth phase. It appears that when the cell density reaches an OD_600_ of 2.5, the secretion of Hcp stops. At the later stationary phase after overnight incubation, when no Hcp was detected, the expression of *hcp* may have been turned off or Hcp may have been proteolytically degraded.

### Temperature-dependent expression and secretion of Hcp by VflT6SS2

**Figure 4:**
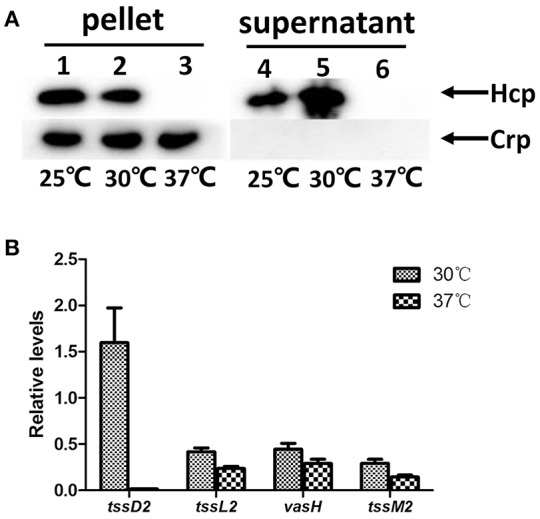
**Effects of temperature on the expression of VflT6SS2 by ***V. fluvialis*** 85003. (A)** Immunoblot analysis of Hcp expression. The *V. fluvialis* strain 85003 was grown in LB containing 170 mM NaCl to an OD_600_ of 1.5 at 25°, 30°, or 37°C. Cell pellets and corresponding supernatants were analyzed by SDS-PAGE and immunoblot assays using anti-Hcp and anti-Crp antisera. Lanes 1–3, cell pellets; lanes 4–6, culture supernatants. The arrows indicate the reaction bands of the Hcp and Crp proteins. **(B)** qRT-PCR analysis of the mRNA abundance of VflT6SS2 genes. The *V. fluvialis* strain 85003 was grown in LB containing 170 mM NaCl to an OD_600_ of 1.5 at 30° or 37°C. RNA was extracted, and the abundances of *tssD*2, *tssL*2, *vasH*, and *tssM*2 mRNA were determined by qRT-PCR as described in the Materials and Methods. Each value is the average for three independent cultures. The error bars indicate standard deviations from the mean.

Like many species of *Vibrios, V. fluvialis* can survive under different environmental conditions, from the aquatic milieu, such as seas, estuaries, and brackish waters, to the human intestine. We wondered whether the temperature affects the function of VflT6SS2 in *V. fluvialis*. Therefore, we cultured the 85003 strain at different temperatures (25°, 30°, or 37°C) and compared the expression and secretion of Hcp. Although 85003 grows faster at 37°C than at 25° and 30°C (Figure S2), the OD_600_ of 1.5 lies at the same log growth phase. Hcp was expressed and secreted at both 25°C and 30°C, but not at 37°C (Figure 4A). The secretion of Hcp at 30°C was higher than that at 25°C (Figure 4A, compare lane 4 with lane 5). Our results revealed that VflT6SS2 is active under cool and warm temperature, similar to T6SS2 in *V. parahaemolyticus*, which is most active at cold (23°C) and warm (30°C) temperatures under low salt conditions (Salomon et al., 2013), suggesting that VflT6SS2 may play a role in the environmental fitness of *V. fluvialis*.

To explore the molecular mechanism(s) responsible for the undetectable protein level of Hcp at 37°C, we measured the transcription levels of *tss*D2 (*hcp*), together with three selected VflT6SS2 core genes [*tssL*2 (*vasF*), *vasH*, and *tssM*2 (*vasK*)] by qRT-PCR using cultures grown at 30° and 37°C. As shown in Figure 4B, the *tss*D2 (*hcp*) mRNA level was dramatically decreased (more than 150-fold) at 37°C compared to that at 30°C. However, no considerable alterations were observed in the mRNA levels of *tssL*2 (*vasF*), *vasH*, and *tssM*2 (*vasK*) between 30° and 37°C, suggesting that the loss of function of VflT6SS2 at 37°C is mainly due to the extremely low transcription level of the *tssD*2 (*hcp*) gene at this temperature.

### Salinity and osmolarity-dependent expression and secretion of Hcp from VflT6SS2

**Figure 5:**
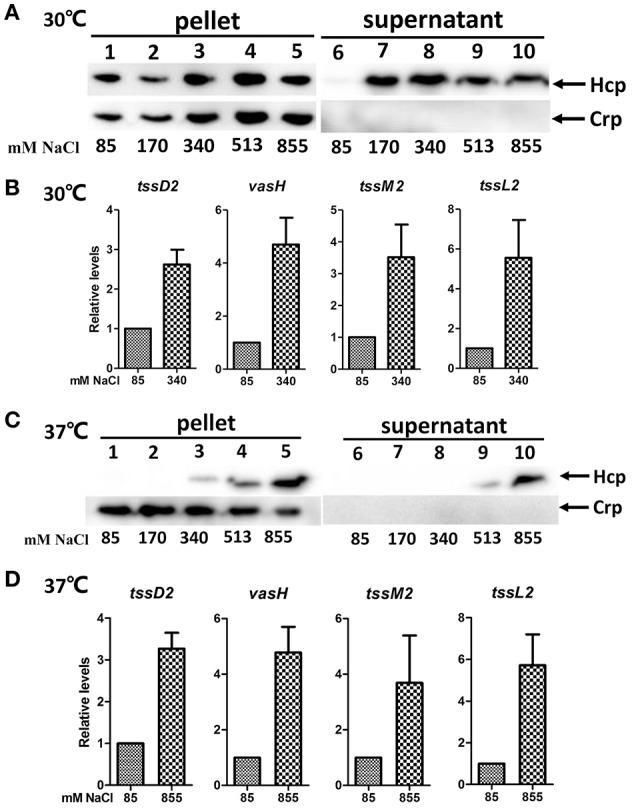
**Effects of salinity on the expression of VflT6SS2 Hcp by ***V. fluvialis*** 85003. (A,C)** Immunoblot analysis of Hcp expression. The *V. fluvialis* strain 85003 was grown at 30° or 37°C in LB containing the indicted concentrations of NaCl. Cell pellets and culture supernatants were analyzed by SDS-PAGE and immunoblot assays using anti-Hcp and anti-Crp antisera. Lanes 1–5, cell pellets; lanes 6–10, culture supernatants. The arrows indicate the reaction bands of the Hcp and Crp proteins. **(B,D)** qRT-PCR analysis of the mRNA abundance of VflT6SS2 genes. The *V. fluvialis* strain 85003 was grown at 30° or 37°C in LB containing the indicted concentrations of NaCl. RNA was extracted, and the mRNA abundances of *tssD*2, *tssL*2, *vasH*, and *tssM*2 were determined by qRT-PCR as described in the Materials and Methods. Data from three independent cultures were plotted to show the relative levels of transcripts, with the average level from low (85 mM NaCl) salinity samples in each case set to 1.0. The error bars indicate standard deviations from the mean.

As *V. fluvialis* is a halophilic species of *Vibrio* and normally resides in coastal and estuarine environments, we investigated if the salinity influences the expression and secretion of Hcp in *V. fluvialis*. We cultured strain 85003 at 30°C to an OD_600_ of 1.5 in LB containing increasing concentrations of NaCl. We observed that comparable amounts of Hcp were detected in the cell pellets regardless of the salt concentration (Figure 5A, left panel). Significant amounts of secreted Hcp were readily detected in supernatants from cultures containing 170, 340, 513, or 855 mM NaCl, but not in supernatants from cultures containing 85 mM NaCl (Figure 5A, right panel, compare lane 6–lanes 7–10), although the secretion levels at 513 and 855 mM NaCl appeared to be slightly reduced compared to those at 170 and 340 mM NaCl. These results indicated that Hcp secretion was stimulated by high NaCl concentrations. To obtain more detailed information, we investigated whether salinity can influence the expression of *tssD*2 and the VflT6SS2 core gene cluster. As shown in Figure 5B, *tssD*2 and the all three selected VflT6SS2 core genes, *tssL*2, *vasH*, and *tssM*2, were apparently induced when strain 85003 was grown at 30°C in LB containing 340 mM NaCl compared to cultures grown with 85 mM NaCl.

We then determined whether the salinity could also affect VflT6SS2 at 37°C. We therefore cultured the 85003 strain at 37°C in LB containing different concentrations of NaCl and analyzed the expression and secretion of Hcp. We found that steadily increasing amounts of Hcp were detected in pellets from cultures supplemented with 340, 513, or 855 mM NaCl (Figure 5C, lanes 3–5), and the secreted form of Hcp was detected under 513 or 855 mM NaCl conditions (Figure 5C, lanes 9–10). On the basis of these results, we inferred that high NaCl concentrations could induce both the expression and secretion of Hcp in a dose-dependent manner at 37°C. Consistently, increased mRNA levels of *tssD*2, *tssL*2, *vasH*, and *tssM*2 were confirmed when the 85003 strain was grown at 37°C in LB containing 855 mM NaCl (Figure 5D). These results indicated that, under certain circumstances, such as high salinity, VflT6SS2 would be activated in an infected host. The precise stimuli and signaling mechanism(s) involved in this response should be further investigated in future studies.

**Figure 6:**
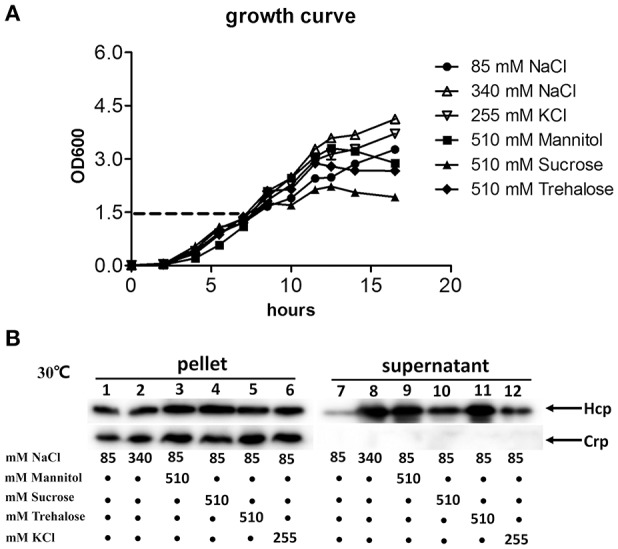
**Effects of osmolarity on the expression and secretion of VflT6SS2 Hcp from ***V. fluvialis*** 85003. (A)** Growth curves of *V. fluvialis* strain 85003 incubated at 30°C in normal LB media (85 mM NaCl) or normal LB supplemented with different amounts of salt (NaCl or KCl) or sugar alcohol (mannitol, sucrose or trehalose) to yield the same osmolarity as 340 mM NaCl. Error bars indicate the standard deviation of three independent cultures. **(B)** Immunoblot analysis of Hcp expression and secretion. The *V. fluvialis* strain 85003 was grown as described above. Lanes 1–6, cell pellets; lanes 7–12, culture supernatants.

In *V. cholerae* A1552, it was reported that Hcp secretion was induced by osmolarity rather than salinity (Ishikawa et al., 2012). To test whether this is also the case in *V. fluvialis*, strain 85003 was grown at 30°C in LB supplemented with mannitol, trehalose or sucrose at a concentration of 510 mM, or KCl at a concentration of 255 mM, in addition to 85 mM NaCl to yield an equivalent osmolarity as 340 mM NaCl. Based on the growth curve (Figure 6A), we collected samples from cultures with an OD_600_ of 1.5. Our results demonstrated that although the protein levels of Hcp in pellets were comparable under low and high osmolarity conditions (Figure 6B, left panel), the protein was more efficiently secreted under high osmolarity conditions (Figure 6B, compare lane 7 with lanes 8–12). Furthermore under the same osmolarity conditions, NaCl, mannitol and trehalose seemed to more potently enhance the secretion of Hcp than sucrose and KCl (Figure 6B, compare lanes 8, 9, 11 with lanes 10 and 12). Together, these results indicate that high osmolarity induces Hcp secretion in *V. fluvialis*, and the osmolarity-dependent secretion of Hcp may be a common characteristic among *Vibrio* species.

### *V. fluvialis* VflT6SS2 plays a role in interbacterial virulence

**Figure 7:**
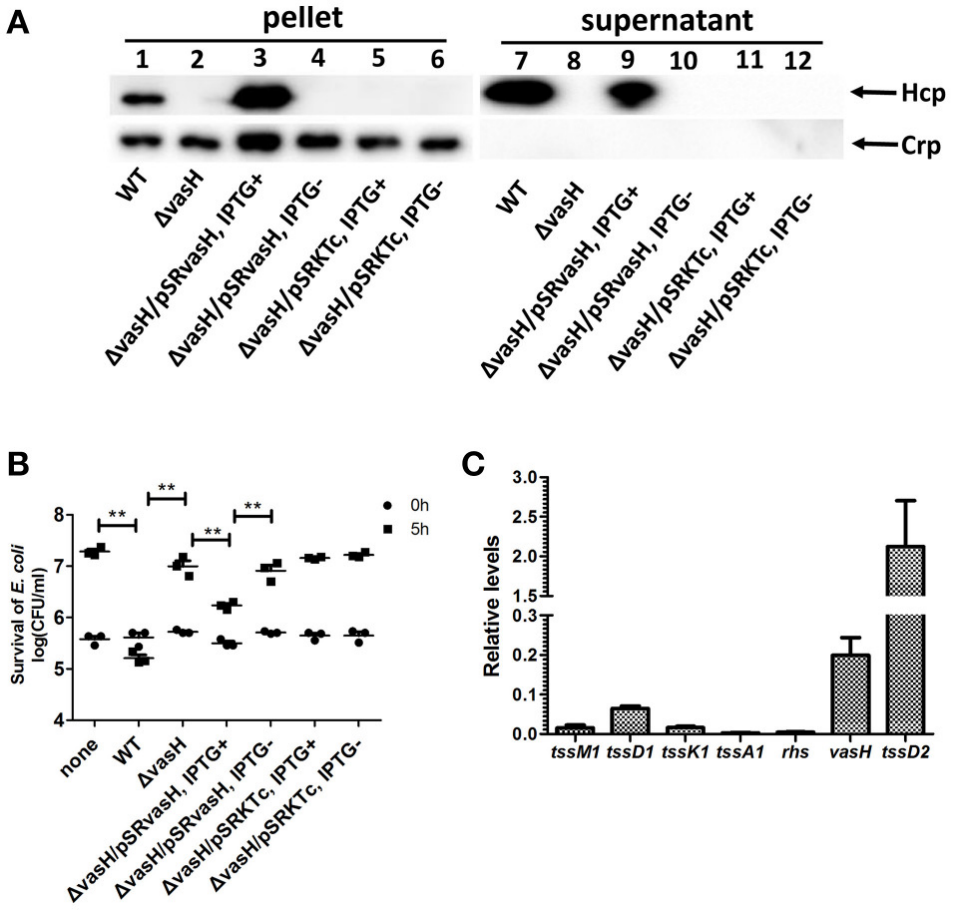
**VflT6SS2-dependent competition between ***V. fluvialis*** 85003 and ***E. coli*** strain MG1655**. **(A)** Immunoblot analysis of Hcp secretion by the *vasH* mutant and the trans-complemented strains. Lanes 1–6, cell pellets; lanes 7–12, culture supernatants. The arrows indicate the reaction bands of the Hcp and Crp proteins. **(B)** The survival of the rifampicin-resistant *E. coli* strain MG1655 was determined after 5 h co-culture with *V. fluvialis* 85003 (WT), Δ*vasH* or the trans-complemented strains at 30°C on LB agar plates containing 340 mM NaCl. The data represent three independent experiments. ^**^,Significant differences between sample groups at 5 h as determined by an unpaired, two-tailed Student's *t*-test (*P* < 0.001). None, medium only; WT, wild type. **(C)** qRT-PCR analysis of the mRNA abundance of selected VflT6SS1 and VflT6SS2 genes. The *V. fluvialis* strain 85003 was grown at 30°C in LB containing 340 mM of NaCl. RNA was extracted, and the mRNA abundances of selected VflT6SS1 genes (*tssM*1, *tssD*1, *tssK*1, *tssA*1, and *rhs*) and VflT6SS2 genes (*vasH* and *tssD*2) were determined by qRT-PCR as described in the Materials and Methods. Each value is the average of three independent cultures. The error bars indicate standard deviations from the mean.

To test if VflT6SS2 activity contributes to interbacterial virulence or competition, we performed an *E. coli* killing assay. We first generated a *vasH* in-frame deletion mutant, Δ*vasH*, which was used as an avirulent control. VasH is considered to be a global transcriptional regulator of T6SS and is required to initiate the transcription of T6SS genes in *V. cholerae* (Kitaoka et al., 2011). The VasH of *V. fluvialis* encodes 531 amino acids and shares 73% identity with that of *V. cholerae*. Of note, the Walker A (GETGTGKE) motif, Walker B (GTLFLDEIG) motif, and the central GAFSGA-loop 1 region of the ATPase domain of VasH are exactly the same in *V. cholerae* and *V. fluvialis* (Figure S3). Consistent with the findings in *V. cholerae*, the deletion of *vasH* in *V. fluvialis* resulted in a lack of expression and secretion of Hcp (Figure 7A, compare lane 1 to lane 2, and lane 7 to lane 8), i.e., the naturally functional VflT6SS2 was completely disabled. However, the expression and secretion of Hcp were restored by introducing a complemented plasmid pSR*vasH* under IPTG induction, but the restoration did not occur if the pSRKTc control plasmid was used or no IPTG induction was employed (Figure 7A). The mixtures of the prey *E. coli* K-12 MG1655 and *V. fluvialis* predators were spotted on LB nutrient agar plates containing 340 mM NaCl and incubated at 30°C. The number of surviving MG1655 was determined after 5 h of incubation. The results showed that the survival of MG1655 was significantly reduced in the presence of wild-type *V. fluvialis*, but not when *vasH* was deleted (Figure 7B). Re-expression of *vasH* from pSR*vasH* recovered the ability of *V. fluvialis* to compete against the *E. coli*, although not to the level of the wild type (Figure 7B). The above results collectively indicate that VflT6SS2 provides *V. fluvialis* with increased competitive fitness.

To determine whether VflT6SS1 is involved in the competitive bacterial killing demonstrated by *V. fluvialis*, we measured the protein level of TssD1, as well as the mRNA levels of three VflT6SS1 genes located at the beginning of the VflT6SS1 gene cluster (*rhs, tssA*1, *tssD*1), and two other genes at its end (*tssK*1 and *tssM*1) using qRT-PCR for bacteria cultured under the same conditions as in the killing assay (340 mM NaCl at 30°C). We could not detect TssD1 expression in either the cell pellet or the supernatant by a Western blot analysis (data not shown). qRT-PCR analyses revealed that the genes selected from the VflT6SS1 cluster showed extremely low mRNA levels (0.015, 0.063, 0.016, 0.003, and 0.004 for *tssM*1, *tssD*1, *tssK*1, *tssA*1, and *rhs*, respectively) compared to *vasH* and *tssD*2 from the VflT6SS2 cluster (0.199 and 2.124 for *vasH* and *tssD*2, respectively; Figure 7C). These results suggested that VflT6SS1 is likely inactive, at least under the tested conditions.

**Figure 8:**
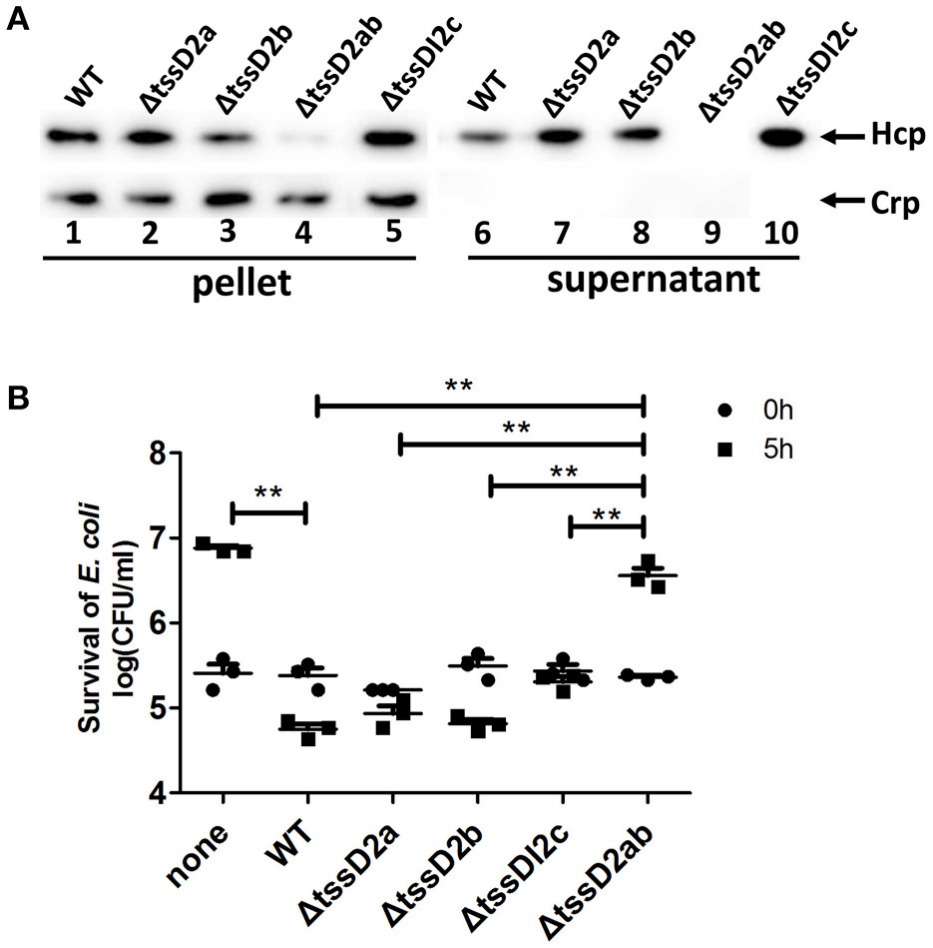
**Different contributions of ***tssD***2_a, ***tssD***2_b, and ***tssD***2_c-***tssI***2_c to the function of VflT6SS2**. **(A)** Immunoblot analysis of Hcp expression in Δ*tssD*2a, Δ*tssD*2b, Δ*tssD*2ab, and Δ*tssDI*2c cultured at 30°C. Lanes 1–5, cell pellets; lanes 6–10, culture supernatants. The arrows indicate the reaction bands of the Hcp and Crp proteins. **(B)** The survival of the rifampicin-resistant *E. coli* strain MG1655 was determined after 5 h of co-culture with *V. fluvialis* 85003 (WT), Δ*tssD*2a, Δ*tssD*2b, Δ*tssD*2ab, or Δ*tssDI*2c at 30°C on LB agar plates containing 340 mM NaCl. The data represent three independent experiments. ^**^,Significant differences between the sample groups at 5 h as determined by an unpaired, two-tailed Student's *t*-test (*P* < 0.05). None, medium only.

Since VflT6SS2 contains three copies of *tssD*2 (namely *tssD*2*_*a, *tssD*2*_*b and *tssD*2*_*c), we generated individual deletion mutants and a *tssD*2*_*ab double-deletion mutant to pinpoint which copies contribute to the function of VflT6SS2. As shown in Figure 8A, deletion of the individual copies (*tssD*2*_*a, *tssD*2*_*b or *tssDI*2*_*c) did not affect the expression or secretion of Hcp, while the combined deletion of *tssD*2a and *tssD*2b completely abolished the secretion of Hcp. Consistently, each deletion mutant of *tssD*2*_*a, *tssD*2*_*b or *tssDI*2*_*c showed similar interbacterial virulence to the wild type organism, but the double mutant (Δ*tssD*2ab) displayed significantly decreased antibacterial virulence (Figure 8B). Based on these results, we speculated that *tssD*2*_*a and *tssD*2*_*b function redundantly and are required for VflT6SS2 to mediate its antibacterial activity, while *tssD*2*_*c is probably dispensable for the formation of VflT6SS2 structural apparatus or the assembly of functional VflT6SS2.

Taken together, these results demonstrate that VflT6SS2 provides an advantage for *V. fluvialis* in competition with bacterial neighbors, which may contribute to its fitness and pathogenesis.

## Discussion

T6SS is a newly identified protein secretion system in Gram-negative bacteria that functions to antagonize the neighboring cells through the delivery of lethal effector molecules. In this study, we investigated the T6SS machinery in *V. fluvialis*, an emerging foodborne pathogen. We showed the detailed genetic contents and organization of two T6SS gene clusters in *V. fluvialis* strain 85003. We found that VflT6SS2 is more widely distributed than VflT6SS1 in different isolates of *V. fluvialis*. VflT6SS2 has three “orphan” *hcp*-*vgrG* clusters and has nearly the same genetic organization and gene orientation as the “core” gene cluster of *V. cholerae* T6SS (VCA0107~VCA0124) except that the homologous counterpart of VCA0122 (*vasM*) was missing from *V. fluvialis* (Figure 1). The third “orphan” *hcp*-*vgrG* cluster, *tssD2_c*-*tssI2_c*, which closely neighbored three predicted phage integrases, was not found in *V. fluvialis* 33809, *V. furnissii* NCTC11218 or *V. cholerae* N16961, implying its unstable characteristics in terms of acquisition and loss.

For the first time, we demonstrated the functional expression of T6SS in *V. fluvialis* (Figure 2). Previous studies have reported that the expression of T6SS is growth phase-dependent (Ishikawa et al., 2009; Pieper et al., 2009). In this study, we confirmed the growth phase-dependent expression of the T6SS and showed that the growth phase regulates the function of VflT6SS2, i.e., the secretion of the Hcp effector is dependent on the growth phase. As shown in Figure 3, the synthesis of Hcp remains stable throughout the growth phase (from an OD_600_ of 0.5 to 3.8; Figure 3A), but Hcp secretion was only observed in a narrow range of the growth phase (from an OD_600_ of 1.0 to 2.0; Figure 3B), suggesting that VflT6SS2 functions only during the late log phase and early stationary phase of the bacterial growth. We know that protein secretion is an energy-consuming process. Turning on the secretion system at certain growth stages may be an adaptive strategy for bacterial cells to avoid unnecessary energy consumption while facilitating cell multiplication and survival. We speculate that this kind of regulation may be achieved through sensing changes in the culture environment, such as the cell density, nutrition consumption, concomitant metabolite accumulation, etc. In support of this hypothesis, it has been reported that quorum sensing, sigma factor RpoN, transcriptional regulators and stress-response-inducing factors activate the T6SS in different bacterial species (Ishikawa et al., 2009; Leung et al., 2011).

Functional expression of VflT6SS2 was activated under cool (25°C) and warm (30°C) temperatures, but was completely repressed at high temperature (37°C), as evidenced by the lack of Hcp detection in both cell pellets and culture supernatants (Figure 4A) and the extremely low transcriptional level of *tssD*2 (Figure 4B). The temperature-dependent expression of T6SS was also reported in other species, including *Vibrio* pathogens such as the *V. cholerae* O1 serogroup (Ishikawa et al., 2012), *V. parahaemolyticus* (Salomon et al., 2013) and even the zoonotic pathogen, *Yersinia pestis* (Pieper et al., 2009). The temperature-specific expression pattern of VflT6SS2, combined with its wide existence in different isolates, suggests that VflT6SS2 probably plays a vital role in *V. fluvialis* bacterial survival and persistence in the environmental niche.

Consistent with what has been reported in *V. cholerae* O1 strain A1552 (Ishikawa et al., 2012), we also observed that osmolarity induced the secretion of Hcp in *V. fluvialis* under the warm temperature (30°C) (Figures 5A, 6B). In addition, a higher NaCl concentration (>340 mM) was observed to induce both the expression and secretion of Hcp in a NaCl concentration-dependent manner at 37°C, a temperature which is usually associated with a lack of Hcp. Furthermore, a clear dose-related response was observed (Figure 5C). Similar to what has reported in *V. cholerae* (Ishikawa et al., 2012), qRT-PCR analyses revealed that high salinity promoted the transcription of *tssD*2 and the VflT6SS2 gene cluster (Figures 5B,D), which may be the main reason for the activation of VflT6SS2. However, other possibilities cannot be excluded, such as an increase in Hcp secretion due to increased contraction of VipA/VipB sheath tubules, which may work more rapidly and efficiently in response to various environmental stimuli. This may be supported by the observation that a high salt concentration improves the stability of the VipA/VipB complex *in vitro* (Bröms et al., 2013). It is also possible that the high salinity affected the dynamic cycle of the sheath tubules (assembly, contraction, disassembly, and reassembly; Basler et al., 2012).

It is worth noting that the range of salinity inducing the secretion and expression of Hcp in *V. cholerae* was much narrower than that in *V. fluvialis*. The presence of NaCl at 595 mM completely abolished the secretion of Hcp in *V. cholerae* (Ishikawa et al., 2012), while in *V. fluvialis*, 855 mM NaCl still efficiently induced Hcp secretion (Figure 5A). We reasoned that this may be because *V. fluvialis* is a halophilic species and is naturally able to tolerate higher salinity than *V. cholerae*. At the same time, the ability to maintain the functional activity of T6SS at a much higher salinity (higher than 513 mM) could provide *V. fluvialis* with a competition advantage in the normal marine habitat, where the NaCl concentration is up to 500 mM (Bröms et al., 2013). *V. fluvialis* was reported to be the predominant pathogenic *Vibrio* species isolated from the final effluents of a rural wastewater treatment plant in South Africa (Igbinosa et al., 2009) and could maintain a long-term survival (6 years) in marine sediment (Amel et al., 2008).

Finally, we showed that WT 85003 exhibited antimicrobial activity when co-cultured with *E. coli* at 30°C (Figure 7B). Deletion of *vasH* compromised the synthesis of Hcp (Figure 7A), and resulted in the loss of bacterial killing activity (Figure 7B). Complementation of *vasH* from the pSR*vasH* plasmid restored the Hcp expression and the bacterial killing activity of the ΔvasH mutant (Figures 7A,B). qRT-PCR analyses confirmed that under the tested bacterial killing conditions, VflT6SS1 was not functional (Figure 7C). Together, these results demonstrated that the antimicrobial activity is VflT6SS2-mediated and requires the expression of both Hcp and the transcriptional regulator, VasH. Although *vasH* is necessary for the functional activity of T6SS, we found that it had the lowest detection rate of the various VflT6SS2 genes in the tested isolates. At present, we do not know whether this is due to sequence variation or gene deletion, and will be investigated in subsequent studies. Furthermore, we investigated the contribution of the three copies of *tssD*2 to the function and interbacterial virulence of VflT6SS2. Our results showed that the deletion of the individual *tssD*2 copies did not significantly affect the interbacterial virulence of VflT6SS2, nor did it affect the expression and secretion of Hcp. In contrast, the deletion of both *tssD*2*_*a and *tssD*2*_*b abolished the expression of Hcp and the interbacterial virulence (Figures 8A,B), suggesting that *tssD*2*_*a and *tssD*2*_*b function redundantly and are required for VflT6SS2 to mediate its antibacterial activity, while *tssDI*2*_*c is probably dispensable, in accordance with its genetic location (i.e., neighbored with phage integrases) and its absence in closely-related *Vibrio* species, such as *V. cholerae, V. furnissii*, and *V. fluvialis* strain 33809 (Figure 1). The redundancy of function of the two *hcp* copies (namely *tssD*2*_*a and *tssD*2*_*b) as structural components and effector proteins of the T6SS was also observed in *V. cholerae* (Ishikawa et al., 2009).

In summary, we obtained evidence that there is at least one functional T6SS in *V. fluvialis* 85003, and that its expression and the conditions leading to its induction are similar to, but distinct from, those of other *Vibrio* species. We hypothesize that VflT6SS2 of *V. fluvialis* constitutes an important factor that facilitates successful competition with other organisms in the marine environment. Our findings are helpful to understand the diversity of the distribution, organization, expression, and regulation of T6SS and further broaden the overall knowledge of the pathogenicity and environmental fitness of *V. fluvialis*.

## Authors contributions

WL and BK conceived and designed the experiments. YH, MZ, WL, BD, and JL performed the experiments. PD and YD performed the bioinformatics analyses. WL, BK, and YH analyzed the data and discussed the results. WL and YH wrote the paper.

### Conflict of interest statement

The authors declare that the research was conducted in the absence of any commercial or financial relationships that could be construed as a potential conflict of interest.

## Supplementary material

Figure S1 **Growth curves of ***V. fluvialis*** strains 85003, VF54, CICC21612, and VF42 incubated in LB media containing 170 mM NaCl at 30°C**. Error bars indicate the standard deviation of three independent cultures.

Figure S2 **Growth curves of ***V. fluvialis*** strain 85003 incubated in LB media containing 170 mM NaCl at 25°, 30°, and 37°C, respectively**. Error bars indicate the standard deviation of three independent cultures.

Figure S3 **Amino acid sequence alignments of the ***V. fluvialis*** 85003 VasH protein (VflVasH) with VasH protein sequences of ***V. cholerae*** (VchVasH) and ***V. furnissii*** (VfuVasH)**. The identical and highly conserved amino acids are highlighted in *black* and *gray*, respectively. The Walker A motif, Walker B motif, the central loop1 region of the ATPase domain and the helix-turn-helix domain are underlined.

## Abbreviations

T6SS: type VI secretion system
Hcp: hemolysin-coregulated protein
VgrG: valine-glycine repeat protein G
LB: Luria-Bertani broth
WT: wild-type
OD600: optical density at 600 nm
PCR: polymerase chain reaction
CFU: colony forming units
ORF: open reading frame
VFH: hemolysin of *V. fluvialis*.

## References

[B1] Ahmed A. M. Shinoda S. Shimamoto T. (2005). A variant type of *Vibrio cholerae* SXT element in a multidrug-resistant strain of *Vibrio fluvialis*. FEMS Microbiol. Lett. 242, 241–247. 10.1016/j.femsle.2004.11.012 15621444

[B2] Allton D. R. Forgione M. A. Jr. Gros S. P. (2006). Cholera-like presentation in Vibrio fluvialis enteritis. South. Med. J. 99, 765–767. 10.1097/01.smj.0000223657.22296.e6 16866063

[B3] Amel B. K. Amine B. Amina B. (2008). Survival of *Vibrio fluvialis* in seawater under starvation conditions. Microbiol. Res. 163, 323–328. 10.1016/j.micres.2006.06.006 16870413

[B4] Aubert D. F. Xu H. Yang J. Shi X. Gao W. Li L.. (2016). A Burkholderia Type VI effector deamidates Rho GTPases to activate the Pyrin Inflammasome and trigger inflammation. Cell Host Microbe 19, 664–674. 10.1016/j.chom.2016.04.004 27133449

[B5] Basler M. Pilhofer M. Henderson G. P. Jensen G. J. Mekalanos J. J. (2012). Type VI secretion requires a dynamic contractile phage tail-like structure. Nature 483, 182–186. 10.1038/nature10846 22367545 PMC3527127

[B6] Bellet J. Klein B. Altieri M. Ochsenschlager D. (1989). *Vibrio fluvialis*, an unusual pediatric enteric pathogen. Pediatr. Emerg. Care 5, 27–28. 2652096 10.1097/00006565-198903000-00008

[B7] Bhattacharjee S. Bhattacharjee S. Bal B. Pal R. Niyogi S. K. Sarkar K. (2010). Is *Vibrio fluvialis* emerging as a pathogen with epidemic potential in coastal region of eastern India following cyclone Aila? J. Health Popul. Nutr. 28, 311–317. 20824973 10.3329/jhpn.v28i4.6036 PMC2965321

[B8] Bingle L. E. Bailey C. M. Pallen M. J. (2008). Type VI secretion: a beginner's guide. Curr. Opin. Microbiol. 11, 3–8. 10.1016/j.mib.2008.01.006 18289922

[B9] Borgeaud S. Metzger L. C. Scrignari T. Blokesch M. (2015). The type VI secretion system of *Vibrio cholerae* fosters horizontal gene transfer. Science 347, 63–67. 10.1126/science.1260064 25554784

[B10] Boyer F. Fichant G. Berthod J. Vandenbrouck Y. Attree I. (2009). Dissecting the bacterial type VI secretion system by a genome wide *in silico* analysis: what can be learned from available microbial genomic resources? BMC Genomics 10:104. 10.1186/1471-2164-10-104 19284603 PMC2660368

[B11] Brenner D. J. Hickman-Brenner F. W. Lee J. V. Steigerwalt A. G. Fanning G. R. Hollis D. G.. (1983). *Vibrio furnissii* (formerly aerogenic biogroup of *Vibrio fluvialis*), a new species isolated from human feces and the environment. J. Clin. Microbiol. 18, 816–824. 6630464 10.1128/jcm.18.4.816-824.1983 PMC270912

[B12] Bröms J. E. Ishikawa T. Wai S. N. Sjöstedt A. (2013). A functional VipA-VipB interaction is required for the type VI secretion system activity of *Vibrio cholerae* O1 strain A1552. BMC Microbiol. 13:96. 10.1186/1471-2180-13-96 23642157 PMC3656785

[B13] Cabrera Rodríguez L. E. Monroy S. P. Morier L. Ramírez Alvarez M. M. Fernández Abreu A. Castro Escarpulli G.. (2005). Severe otitis due to Vibrio fluvialis in a patient with AIDs: first report in the world. Rev. Cubana Med. Trop. 57, 154–155. 17966587

[B14] Chikahira M. Hamada K. (1988). Enterotoxigenic substance and other toxins produced by *Vibrio fluvialis* and *Vibrio furnissii*. Nippon. Juigaku Zasshi 50, 865–873. 3172595 10.1292/jvms1939.50.865

[B15] Chowdhury G. Pazhani G. P. Dutta D. Guin S. Dutta S. Ghosh S.. (2012). Vibrio fluvialis in Patients with Diarrhea, Kolkata, India. Emerg. Infect. Dis. 18, 1868–1871. 10.3201/eid1811.120520 23092520 PMC3559161

[B16] Chowdhury G. Pazhani G. P. Nair G. B. Ghosh A. Ramamurthy T. (2011). Transferable plasmid-mediated quinolone resistance in association with extended-spectrum beta-lactamases and fluoroquinolone-acetylating aminoglycoside-6′-N-acetyltransferase in clinical isolates of *Vibrio fluvialis*. Int. J. Antimicrob. Agents 38, 169–173. 21683552 10.1016/j.ijantimicag.2011.04.013

[B17] Dong T. G. Ho B. T. Yoder-Himes D. R. Mekalanos J. J. (2013). Identification of T6SS-dependent effector and immunity proteins by Tn-seq in *Vibrio cholerae*. Proc. Natl. Acad. Sci. U.S.A. 110, 2623–2628. 10.1073/pnas.1222783110 23362380 PMC3574944

[B18] Han J. H. Lee J. H. Choi Y. H. Park J. H. Choi T. J. Kong I. S. (2002). Purification, characterization and molecular cloning of Vibrio fluvialis hemolysin. Biochim. Biophys. Acta 1599, 106–114. 12479411 10.1016/s1570-9639(02)00407-7

[B19] Ho B. T. Dong T. G. Mekalanos J. J. (2014). A view to a kill: the bacterial type VI secretion system. Cell Host Microbe 15, 9–21. 10.1016/j.chom.2013.11.008 24332978 PMC3936019

[B20] Huang K. C. Hsu R. W. (2005). *Vibrio fluvialis* hemorrhagic cellulitis and cerebritis. Clin. Infect. Dis. 40, e75–e77. 10.1086/429328 15825019

[B21] Huq M. I. Alam A. K. Brenner D. J. Morris G. K. (1980). Isolation of Vibrio-like group, EF-6, from patients with diarrhea. J. Clin. Microbiol. 11, 621–624. 7430332 10.1128/jcm.11.6.621-624.1980 PMC273473

[B22] Igbinosa E. O. Obi L. C. Okoh A. I. (2009). Occurrence of potentially pathogenic vibrios in final effluents of a wastewater treatment facility in a rural community of the Eastern Cape Province of South Africa. Res. Microbiol. 160, 531–537. 10.1016/j.resmic.2009.08.007 19732825

[B23] Igbinosa E. O. Okoh A. I. (2010). *Vibrio fluvialis*: an unusual enteric pathogen of increasing public health concern. Int. J. Environ. Res. Public Health 7, 3628–3643. 10.3390/ijerph7103628 21139853 PMC2996184

[B24] Ishikawa T. Rompikuntal P. K. Lindmark B. Milton D. L. Wai S. N. (2009). Quorum sensing regulation of the two hcp alleles in *Vibrio cholerae* O1 strains. PLoS ONE 4:e6734. 10.1371/journal.pone.0006734 19701456 PMC2726435

[B25] Ishikawa T. Sabharwal D. Bröms J. Milton D. L. Sjöstedt A. Uhlin B. E.. (2012). Pathoadaptive conditional regulation of the type VI secretion system in *Vibrio cholerae* O1 strains. Infect. Immun. 80, 575–584. 10.1128/IAI.05510-11 22083711 PMC3264300

[B26] Khan S. R. Gaines J. Roop R. M. II. Farrand S. K. (2008). Broad-host-range expression vectors with tightly regulated promoters and their use to examine the influence of TraR and TraM expression on Ti plasmid quorum sensing. Appl. Environ. Microbiol. 74, 5053–5062. 10.1128/AEM.01098-08 18606801 PMC2519271

[B27] Kitaoka M. Miyata S. T. Brooks T. M. Unterweger D. Pukatzki S. (2011). VasH is a transcriptional regulator of the type VI secretion system functional in endemic and pandemic *Vibrio cholerae*. J. Bacteriol. 193, 6471–6482. 10.1128/JB.05414-11 21949076 PMC3232897

[B28] Klontz K. C. Desenclos J. C. (1990). Clinical and epidemiological features of sporadic infections with *Vibrio fluvialis* in Florida, USA. J. Diarrhoeal Dis. Res. 8, 24–26. 2229987

[B29] Kothary M. H. Lowman H. McCardell B. A. Tall B. D. (2003). Purification and characterization of enterotoxigenic El Tor-like hemolysin produced by *Vibrio fluvialis*. Infect. Immun. 71, 3213–3220. 12761101 10.1128/IAI.71.6.3213-3220.2003 PMC155747

[B30] Lai C. H. Hwang C. K. Chin C. Lin H. H. Wong W. W. Liu C. Y. (2006). Severe watery diarrhoea and bacteraemia caused by *Vibrio fluvialis*. J. Infect. 52, e95–e98. 10.1016/j.jinf.2005.05.023 15996742

[B31] Lee J. V. Shread P. Furniss A. L. Bryant T. N. (1981). Taxonomy and description of *Vibrio fluvialis* sp. nov. (synonym group *F. vibrios*, group EF6). J. Appl. Bacteriol. 50, 73–94. 6971864 10.1111/j.1365-2672.1981.tb00873.x

[B32] Leiman P. G. Basler M. Ramagopal U. A. Bonanno J. B. Sauder J. M. Pukatzki S.. (2009). Type VI secretion apparatus and phage tail-associated protein complexes share a common evolutionary origin. Proc. Natl. Acad. Sci. U.S.A. 106, 4154–4159. 10.1073/pnas.0813360106 19251641 PMC2657435

[B33] Leung K. Y. Siame B. A. Snowball H. Mok Y. K. (2011). Type VI secretion regulation: crosstalk and intracellular communication. Curr. Opin. Microbiol. 14, 9–15. 10.1016/j.mib.2010.09.017 20971679

[B34] Liang P. Cui X. Du X. Kan B. Liang W. (2013). The virulence phenotypes and molecular epidemiological characteristics of *Vibrio fluvialis* in China. Gut Pathog. 5:6. 10.1186/1757-4749-5-6 23522652 PMC3636005

[B35] Liu L. Hao S. Lan R. Wang G. Xiao D. Sun H.. (2015). The Type VI secretion system modulates Flagellar gene expression and secretion in *Citrobacter freundii* and contributes to adhesion and cytotoxicity to Host Cells. Infect. Immun. 83, 2596–2604. 10.1128/IAI.03071-14 25870231 PMC4468558

[B36] Liu W. L. Chiu Y. H. Chao C. M. Hou C. C. Lai C. C. (2011). Biliary tract infection caused by Vibrio fluvialis in an immunocompromised patient. Infection 39, 495–496. 10.1007/s15010-011-0146-0 21710120

[B37] Lockwood D. E. Kreger A. S. Richardson S. H. (1982). Detection of toxins produced by vibrio fluvialis. Infect. Immun. 35, 702–708. 7035373 10.1128/iai.35.2.702-708.1982 PMC351098

[B38] Lu X. Liang W. Wang Y. Xu J. Zhu J. Kan B. (2014). Identification of genetic bases of *Vibrio fluvialis* species-specific biochemical pathways and potential virulence factors by comparative genomic analysis. Appl. Environ. Microbiol. 80, 2029–2037. 10.1128/AEM.03588-13 24441165 PMC3957645

[B39] Miyoshi S. Sonoda Y. Wakiyama H. Rahman M. M. Tomochika K. Shinoda S.. (2002). An exocellular thermolysin-like metalloprotease produced by Vibrio fluvialis: purification, characterization, and gene cloning. Microb. Pathog. 33, 127–134. 12220989 10.1006/mpat.2002.0520

[B40] Mougous J. D. Cuff M. E. Raunser S. Shen A. Zhou M. Gifford C. A.. (2006). A virulence locus of *Pseudomonas aeruginosa* encodes a protein secretion apparatus. Science 312, 1526–1530. 10.1126/science.1128393 16763151 PMC2800167

[B41] Pieper R. Huang S. T. Robinson J. M. Clark D. J. Alami H. Parmar P. P.. (2009). Temperature and growth phase influence the outer-membrane proteome and the expression of a type VI secretion system in *Yersinia pestis*. Microbiology 155(Pt 2), 498–512. 10.1099/mic.0.022160-0 19202098

[B42] Pukatzki S. Ma A. T. Sturtevant D. Krastins B. Sarracino D. Nelson W. C.. (2006). Identification of a conserved bacterial protein secretion system in *Vibrio cholerae* using the Dictyostelium host model system. Proc. Natl. Acad. Sci. U.S.A. 103, 1528–1533. 10.1073/pnas.0510322103 16432199 PMC1345711

[B43] Ratnaraja N. Blackmore T. Byrne J. Shi S. (2005). *Vibrio fluvialis* peritonitis in a patient receiving continuous ambulatory peritoneal dialysis. J. Clin. Microbiol. 43, 514–515. 10.1128/JCM.43.1.514-515.2005 15635032 PMC540184

[B44] Salomon D. Gonzalez H. Updegraff B. L. Orth K. (2013). *Vibrio parahaemolyticus* type VI secretion system 1 is activated in marine conditions to target bacteria, and is differentially regulated from system 2. PLoS ONE 8:e61086. 10.1371/journal.pone.0061086 23613791 PMC3628861

[B45] Salomon D. Klimko J. A. Trudgian D. C. Kinch L. N. Grishin N. V. Mirzaei H.. (2015). Type VI secretion system toxins horizontally shared between marine bacteria. PLoS Pathog. 11:e1005128. 10.1371/journal.ppat.1005128 26305100 PMC4549250

[B46] Schell M. A. Ulrich R. L. Ribot W. J. Brueggemann E. E. Hines H. B. Chen D.. (2007). Type VI secretion is a major virulence determinant in *Burkholderia mallei*. Mol. Microbiol. 64, 1466–1485. 10.1111/j.1365-2958.2007.05734.x 17555434

[B47] Shalom G. Shaw J. G. Thomas M. S. (2007). *In vivo* expression technology identifies a type VI secretion system locus in *Burkholderia pseudomallei* that is induced upon invasion of macrophages. Microbiology 153(Pt 8), 2689–2699. 10.1099/mic.0.2007/006585-0 17660433

[B48] Sheng L. Gu D. Wang Q. Liu Q. Zhang Y. (2012). Quorum sensing and alternative sigma factor RpoN regulate type VI secretion system I (T6SSVA1) in fish pathogen *Vibrio alginolyticus*. Arch. Microbiol. 194, 379–390. 10.1007/s00203-011-0780-z 22173829

[B49] Shneider M. M. Buth S. A. Ho B. T. Basler M. Mekalanos J. J. Leiman P. G. (2013). PAAR-repeat proteins sharpen and diversify the type VI secretion system spike. Nature 500, 350–353. 10.1038/nature12453 23925114 PMC3792578

[B50] Song L. Huang Y. Zhao M. Wang Z. Wang S. Sun H.. (2015). A critical role for hemolysin in *Vibrio fluvialis*-induced IL-1beta secretion mediated by the NLRP3 inflammasome in macrophages. Front. Microbiol. 6:510. 10.3389/fmicb.2015.00510 26052324 PMC4440915

[B51] Srinivasan V. B. Virk R. K. Kaundal A. Chakraborty R. Datta B. Ramamurthy T.. (2006). Mechanism of drug resistance in clonally related clinical isolates of Vibrio fluvialis isolated in Kolkata, India. Antimicrob. Agents Chemother. 50, 2428–2432. 10.1128/AAC.01561-05 16801422 PMC1489780

[B52] Suarez G. Sierra J. C. Sha J. Wang S. Erova T. E. Fadl A. A.. (2008). Molecular characterization of a functional type VI secretion system from a clinical isolate of *Aeromonas hydrophila*. Microb. Pathog. 44, 344–361. 10.1016/j.micpath.2007.10.005 18037263 PMC2430056

[B53] Wang Y. Wang H. Liang W. Hay A. J. Zhong Z. Kan B.. (2013). Quorum sensing regulatory cascades control *Vibrio fluvialis* pathogenesis. J. Bacteriol. 195, 3583–3589. 10.1128/JB.00508-13 23749976 PMC3754567

[B54] Weber B. Hasic M. Chen C. Wai S. N. Milton D. L. (2009). Type VI secretion modulates quorum sensing and stress response in *Vibrio anguillarum*. Environ. Microbiol. 11, 3018–3028. 10.1111/j.1462-2920.2009.02005.x 19624706

[B55] Williams S. G. Varcoe L. T. Attridge S. R. Manning P. A. (1996). *Vibrio cholerae* Hcp, a secreted protein coregulated with HlyA. Infect. Immun. 64, 283–289. 8557353 10.1128/iai.64.1.283-289.1996 PMC173757

[B56] Wu C. F. Lin J. S. Shaw G. C. Lai E. M. (2012). Acid-induced type VI secretion system is regulated by ExoR-ChvG/ChvI signaling cascade in *Agrobacterium tumefaciens*. PLoS Pathog. 8:e1002938. 10.1371/journal.ppat.1002938 23028331 PMC3460628

[B57] Wu R. Zhao M. Li J. Gao H. Kan B. Liang W. (2015). Direct regulation of the natural competence regulator gene tfoX by cyclic AMP (cAMP) and cAMP receptor protein (CRP) in Vibrios. Sci. Rep. 5:14921. 10.1038/srep14921 26442598 PMC4595672

[B58] Yu Y. Yang H. Li J. Zhang P. Wu B. Zhu B.. (2012). Putative type VI secretion systems of *Vibrio parahaemolyticus* contribute to adhesion to cultured cell monolayers. Arch. Microbiol. 194, 827–835. 10.1007/s00203-012-0816-z 22535222

[B59] Zheng J. Leung K. Y. (2007). Dissection of a type VI secretion system in *Edwardsiella tarda*. Mol. Microbiol. 66, 1192–1206. 10.1111/j.1365-2958.2007.05993.x 17986187

