# Supplementary figures and images for: Functional Characterization and Conditional Regulation of the Type VI Secretion System in Vibrio fluvialis
Source: Front Microbiol. 2017 Mar 30;8:528. doi: 10.3389/fmicb.2017.00528 (PMC5371669; doi:10.3389/fmicb.2017.00528)

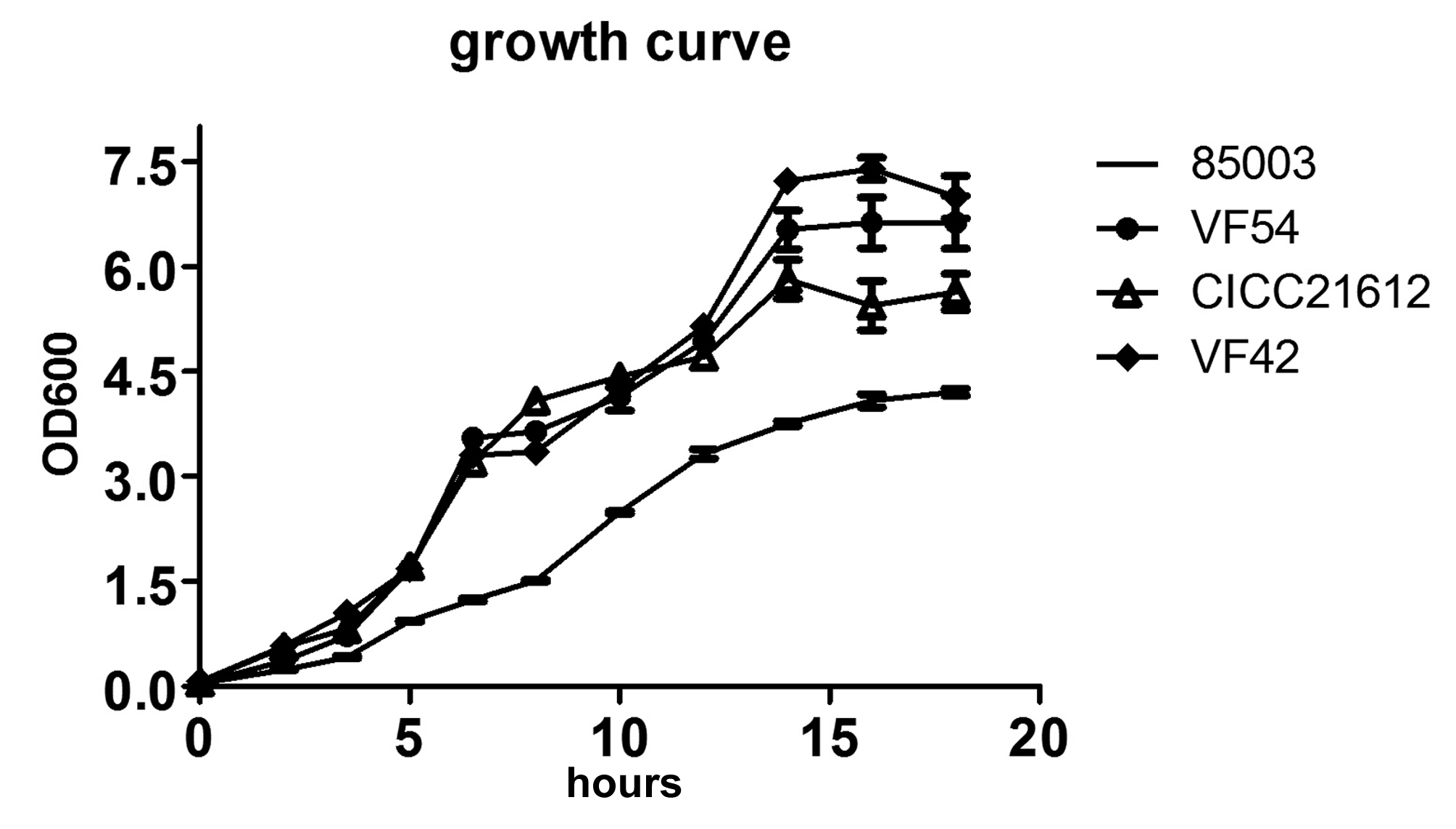

Supplement: Figure S1 — Growth curves of V. fluvialis strains 85003, VF54, CICC21612, and VF42 incubated in LB media containing 170 mM NaCl at 30°C. Error bars indicate the standard deviation of three independent cultures. [file Image1.JPEG]

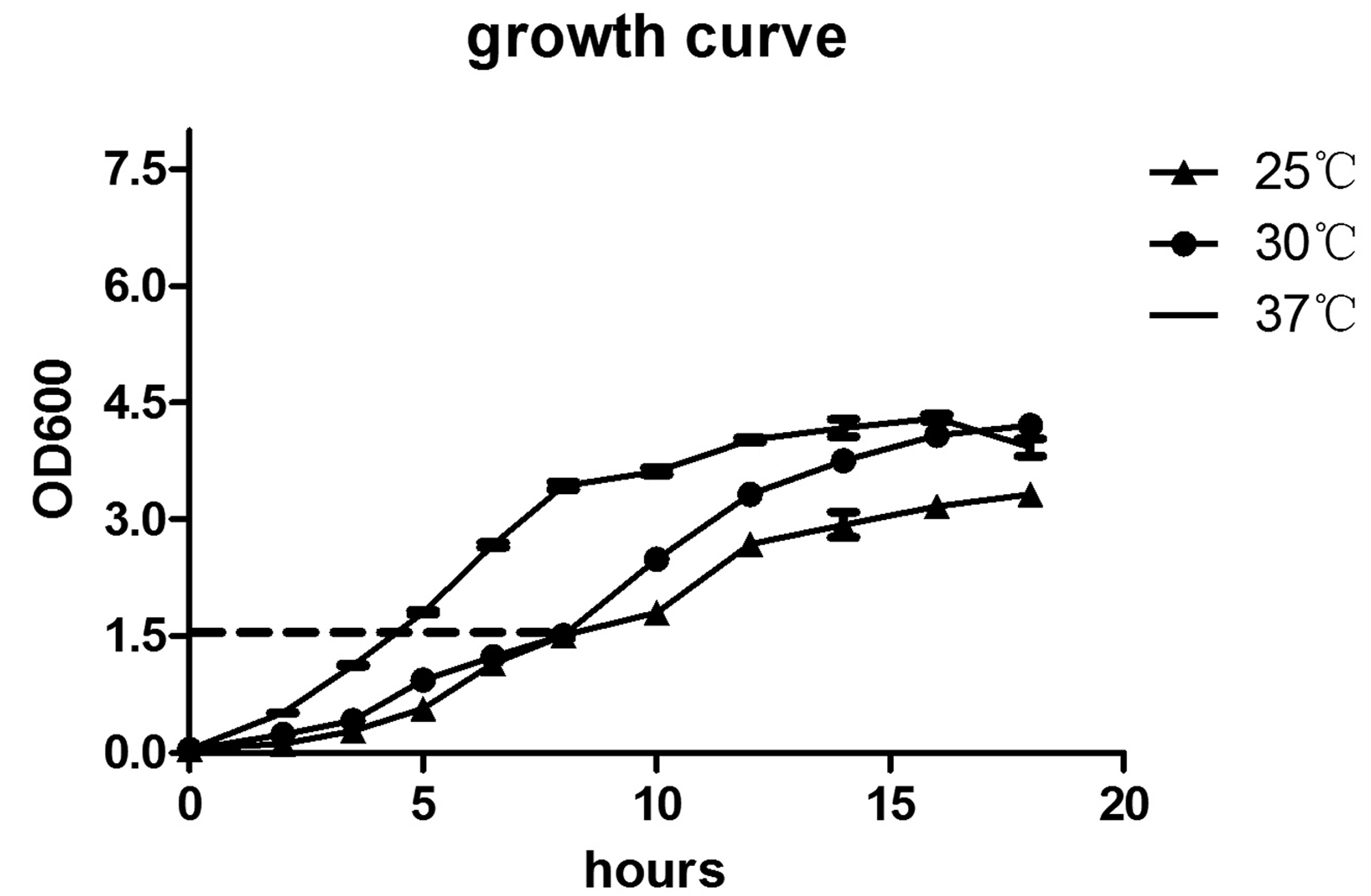

Supplement: Figure S2 — Growth curves of V. fluvialis strain 85003 incubated in LB media containing 170 mM NaCl at 25°, 30°, and 37°C, respectively. Error bars indicate the standard deviation of three independent cultures. [file Image2.JPEG]

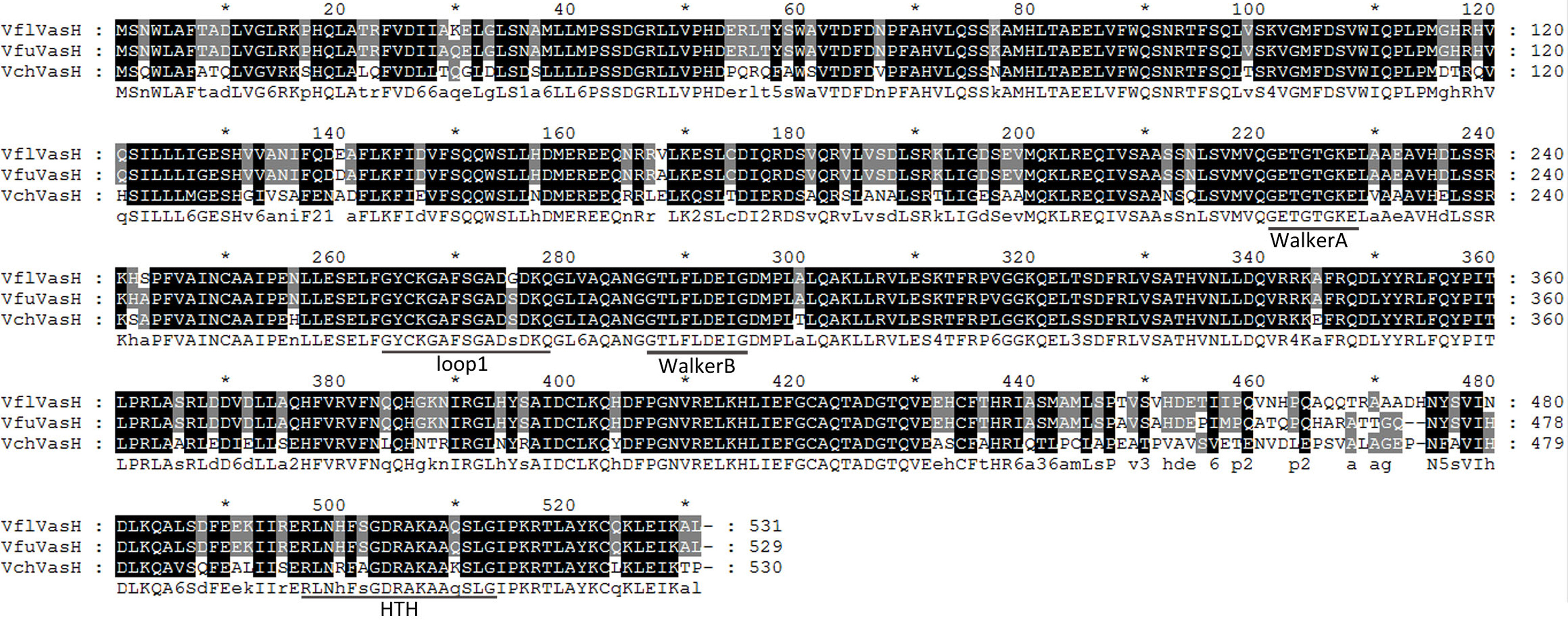

Supplement: Figure S3 — Amino acid sequence alignments of the V. fluvialis 85003 VasH protein (VflVasH) with VasH protein sequences of V. cholerae (VchVasH) and V. furnissii (VfuVasH). The identical and highly conserved amino acids are highlighted in black and gray, respectively. The Walker A motif, Walker B motif, the central loop1 region of the ATPase domain and the helix-turn-helix domain are underlined. [file Image3.JPEG]
